# Two Similar Signatures for Predicting the Prognosis and Immunotherapy Efficacy of Stomach Adenocarcinoma Patients

**DOI:** 10.3389/fcell.2021.704242

**Published:** 2021-08-03

**Authors:** Taohua Yue, Shuai Zuo, Jing Zhu, Shihao Guo, Zhihao Huang, Jichang Li, Xin Wang, Yucun Liu, Shanwen Chen, Pengyuan Wang

**Affiliations:** Division of General Surgery, Peking University First Hospital, Peking University, Beijing, China

**Keywords:** stomach adenocarcinoma, macrophage abundance, 3-gene signature, prognosis, immunotherapy efficacy

## Abstract

**Background:**

Globally, stomach adenocarcinoma (STAD)’s high morbidity and mortality should arouse our urgent attention. How long can STAD patients survive after surgery and whether novel immunotherapy is effective are questions that our clinicians cannot escape.

**Methods:**

Various R packages, GSEA software, Metascape, STRING, Cytoscape, Venn diagram, TIMER2.0 website, TCGA, and GEO databases were used in our study.

**Results:**

In the TCGA and GEO, macrophage abundance of STAD tissues was significantly higher than that of adjacent tissues and was an independent prognostic factor, significantly related to the overall survival (OS) of STAD patients. Between the high- and low- macrophage abundance, we conducted differential expression, univariate and multivariate Cox analysis, and obtained 12 candidate genes, and finally constructed a 3-gene signature. Both low macrophage abundance group and group D had higher TMB and PD-L1 expression. Furthermore, top 5 common gene-mutated STAD tissues had lower macrophage abundance. Macrophage abundance and 3 key genes expression were also lower in the Epstein-Barr Virus (EBV) and HM-indel STAD subtypes and significantly correlated with the tumor microenvironment score. The functional enrichment and ssGSEA revealed 2 signatures were similar and closely related to BOQUEST_STEM_CELL_UP, including genes up-regulated in proliferative stromal stem cells. Hsa-miR-335-5p simultaneously regulated 3 key genes and significantly related to the expression of PD-L1, CD8A and PDCD1.

**Conclusion:**

macrophage abundance and 3-gene signature could simultaneously predict the OS and immunotherapy efficacy, and both 2 signatures had remarkable similarities. Hsa-miR-335-5p and BOQUEST_STEM_CELL_UP might be novel immunotherapy targets.

## Introduction

According to the Global Cancer Statistics 2020, the incidence and mortality of stomach adenocarcinoma (STAD) rank fifth and fourth, respectively (Sung et al., 2021). So far, the histology grades and TNM stages in the guidelines are still the primary standards for evaluating the prognosis of STAD patients (Salati et al., 2019). Glasgow Prognostic Score (GPS) that have not been included in the guidelines can be used as independent indicators for prognostic prediction of STAD patients without perioperative treatment (Melling et al., 2016). However, there are no satisfactory results in predicting the prognosis of STAD patients. Therefore, it is urgent to determine the potential factors for predicting prognosis in STAD.

Currently, the curative efficacies of comprehensive clinical treatments including surgery, radiotherapy, and chemotherapy, are not satisfactory (Kang et al., 2012). Molecularly targeted therapies, such as the human epidermal growth factor receptor 2 (HER2) monoclonal antibody, can improve the prognosis of advanced STAD. Still, they are only suitable for a small number of STAD patients and face the problem of drug resistance at the same time (Bang et al., 2010). Moreover, although PD-1/PD-L1 immune checkpoint inhibitors have opened up new ways to treat STAD patients (Kang et al., 2017), the positive response to immunotherapy was also limited to a small number of STAD patients, and the existing predictors of immunotherapy efficacy need to be improved (Salati et al., 2019).

In summary, we urgently needed an emerging method that could not only assess the prognosis of STAD patients, but also screen STAD patients suitable for immunotherapy.

In our research, compared with adjacent tissues, macrophage abundance was significantly increased in STAD tissues. In terms of prognosis, macrophage abundance was significantly related to the overall survival (OS) of STAD patients. Furthermore, the univariate and multivariate Cox analysis revealed that macrophage abundance was an independent prognostic factor for STAD patients. The higher the abundance of macrophages, the worse the prognosis of STAD patients. In the aspect of predicting the efficacy of immunotherapy, the low macrophage abundance group had a higher tumor mutational burden (TMB) and more PD-L1 (CD274) expression. Among the 6 most common gene mutations in the STAD, macrophage abundance in TTN/TP53/MUC16/LRP1B/SYNE1-mutant tissues was significantly lower than that of wild-type (WT) tissues.

To facilitate clinical quantification of macrophage abundance, based on the macrophage abundance of TCGA and GSE26899 data sets, we performed differential gene expression, univariate and multivariate Cox analysis, and finally obtained 12 candidate genes. With the help of the TIMER2.0 website, Kaplan-Meier (K-M), and Spearman correlation analysis, we identified 3 macrophage-related genes, including ABCA8, LUM, and SHC4 and further built a 3-gene signature. For convenience, we named them 3 key genes.

Among 5 molecular subtypes, Epstein-Barr virus (EBV) and HM-indel subtypes had the lowest macrophage abundance, 3 key genes expression, and the highest PD-L1 expression. The low macrophage group and group D had a higher proportion of EBV and HM-indel subtypes. Above results proved that these 2 subtypes were most suitable for immunotherapy.

Tumor purity as a prognosis and immunotherapy relevant feature in gastric cancer. We scored the stromal and immune components of tumor microenvironment (TME), and macrophage abundance and 3 key genes were all significantly negatively correlated with tumor purity and positively correlated with stromal scores. The 3-gene signature and macrophage abundance had similarities in predicting the OS, immunotherapy efficacy, and Gene Set Enrichment Analysis (GSEA).

To mine the molecular mechanisms of macrophage abundance and 3-gene signature in prognosis and immunotherapy efficacy prediction, we conducted functional enrichment analysis of 721 differentially expressed genes and 36 highly correlated genes, respectively. The up-regulated genes in proliferative stromal stem cells might be related to the above 2 signatures. For the regulatory network of microRNAs and RNA binding proteins of 3 key genes, on the Encyclopedia of RNA Interactomes (ENCORI) website, we unearthed 1 shared microRNAs and 8 RNA binding proteins (RBPs). Hsa-miR-335-5p was significantly related to PD-L1, CD8A and PDCD1.

In conclusion, we had built 2 similar signatures to evaluate the prognosis and immunotherapy efficacy of STAD patients. In terms of practicality, the 3-gene signature is more suitable for clinical application. Hsa-miR-335-5p and BOQUEST_STEM_CELL_UP might be novel immunotherapy targets that needed more verification.

## Materials and Methods

### Ethics Statement

All data in our study were obtained from online public databases and did not involve any *in vitro* or *in vivo* experiments.

### Study Samples

Specifically, 407, 357, 134, and 96 GC patients from the TCGA, GSE84433 (Yoon et al., 2020), GSE29272 (Wang et al., 2013), and GSE26899 (Oh et al., 2018), were enrolled in our study, respectively. Patients with a follow-up time <30 days or incomplete clinical information were excluded from the study. The detailed information was described in Supplementary Table 1. For normalization, the counts data of all genes in each sample of the TCGA database were transformed to transcripts per million (TPM) values for all the samples (Vera Alvarez et al., 2019).

### Quantification of Immune Cell Infiltrations

The TIMER2.0^1^ uses 6 kinds of the most advanced algorithms, including TIMER, CIBERSORT, quanTIseq, xCell, MCP counter, and EPIC algorithms, and provides a more reliable estimate of the immune infiltration level for the Cancer Genome Atlas (TCGA) or tumor atlas provided by users (Li et al., 2020c). Besides, the TIMER2.0 also provides comprehensive analysis and visualization functions of tumor-infiltrating immune cells (Li et al., 2017). Based on gene expression matrix in the TCGA and GEO, the TIMER2.0 was used to quantify immune cell abundance both in STAD and normal samples. In the TCGA and GSE84433 data sets, based on the respective median macrophage abundance, we divided STAD patients into two groups, high and low macrophage infiltration groups. Then, we explored the associations between 12 candidate genes and clinical outcomes, 3 key genes and macrophage abundance in the pan-cancer on the TIMER2.0 website.

### Prognostic Analysis

Based on “survival” and “survminer” packages, Kaplan–Meier (K–M) analysis and log-rank test were performed to evaluate the differences in the overall survival (OS) of STAD patients and establish survival curves (Li et al., 2020b). Furthermore, univariate and multivariate Cox regression analysis were used to identify independent prognostic factors and macrophage-related prognostic genes. *P* < 0.05 was set as the cut-off value.

### Differential Expression Analysis

The Bioconductor packages ‘‘DESeq2’’^2^ (Love et al., 2014) (TCGA data set) and ‘‘limma’’^3^ (Ritchie et al., 2015) (GEO data set) were used to identify differentially expressed genes (DEGs) between high and low macrophage groups, group A and group D, with the criteria of | log_2_(fold-change)| > 1 and false discovery rate < 0.05.

### The ONCOMINE Database

The ONCOMINE database^4^ is a friendly pan-cancer database that integrates RNA- and DNA-seq data from multiple sources, including GEO, TCGA, and published literature, designed to facilitate discovery from genome-wide expression analysis (Rhodes et al., 2004). The filter parameters were set as follows: *P*-value: 1E-4, Fold Change: 2, Gene Rank: Top 10%, Data Type: All.

### Mutation Analysis

The TCGA mutation data were processed and visualized by R package “maftools” (Mayakonda et al., 2018).

### Gene Set Enrichment Analysis (GSEA)

The “sva” package was used to remove batch effects and other unwanted variation in high-throughput data (Leek et al., 2012). In TCGA and GSE84433 joint data, to determine the significantly altered KEGG pathways and hallmark biological states or processes, we performed GSEA between high and low macrophage abundance, 3 key genes, group A and group D, using GSEA software 4.0.1. The (c2.cp.kegg.v7.2.symbols.gmt and h.all.v7.2.symbols.gmt) files were downloaded as the reference gene sets. The nominal (NOM) *P*-value < 0.05 and FDR *q*-value < 0.25 were set as the cut-off criteria.

### UALCAN

The UALCAN^5^ is a comprehensive, user-friendly, and interactive web resource for analyzing cancer OMICS data of TCGA and CPTAC (Chandrashekar et al., 2017). With the help of UALCAN, we analyzed mRNA expression levels of 3 key genes in normal tissues and different cancer stages or grades. The significant difference threshold was set to *P* < 0.05.

### The cBioPortal for Cancer Genomics

The cBioPortal for Cancer Genomics^6^ is an open-access resource for interactive exploration of multiple cancer genomics data sets, whose data derived from TCGA, ICGC, GEO, and other databases. The types of integrated genomic data include somatic mutations, DNA copy number changes (CNAs), mRNA and miRNA expression, DNA methylation, protein abundance, and phosphoprotein abundance (Cerami et al., 2012; Gao et al., 2013). Through this database, we obtained somatic mutation data of TCGA-STAD.

### Expression Profiles of Predictive Biomarkers in Cancer Immunotherapy

In this study, based on non-parametric Mann–Whitney test, we quantified the expression differences of several key predictive biomarkers in cancer immunotherapy [Tumor Mutation Burden (TMB), PD-L1, GZMA, GZMB, PRF1, EOMES, IFNG, TNF, CXCL9, CXCL10, CD8A, CD4, FOXP3, ICOS, CTLA4, LAG3, CD276, VTCN1, CD70, HAVCR2, CD40, CD47, TNFRSF18, TIGIT, TNFSF14, ICAM1, and IL6] between high- and low-macrophage groups, and group A–D. *P* < 0.05 was considered to indicate a statistically significant difference.

### Molecular Subtypes of TCGA-STAD

On the UCSC xena website, we mined the molecular subtypes of TCGA-STAD. Specifically, it included 5 molecular subtypes (GS, CIN, HM-indel, HM-SNV, and EBV) (version2017-06-25) (Liu et al., 2018).

### The Tumor Microenvironment (TME) Score

An algorithm called ESTIMATE (Estimation of STromal and Immune cells in Malignant Tumor tissues using Expression data) was used for the estimation of stromal and immune cells in malignant tumor tissues based on the expression data. Stromal score represented the presence of stroma in tumor tissue, immune score captured the infiltration of immune cells in tumor tissue, and ESTIMATE score infers tumor purity (Yoshihara et al., 2013).

### The Functional Enrichment Analysis

The Metascape^7^ is a reliable, friendly tool for functional enrichment analysis, which integrates multiple databases, such as GO, KEGG, UniProt, and DrugBank (Zhou et al., 2019). The thresholds were set as follows: the number of min overlap and min enrichment were 3, and the P-value cutoff was 0.01.

### The Single Sample Gene Set Enrichment Analysis (ssGSEA)

In our study, the ssGSEA was performed by package “gsva” (Hänzelmann et al., 2013), which was applied to the transcriptome of STAD samples to identify the association between 3 key genes, macrophage abundance and BOQUEST_STEM_CELL_UP score.

### MicroRNA (miRNA) and RNA Binding Proteins (RBPs) Networks

On the ENCORI^8^ and TarBase v.8^9^, we obtains the microRNAs (miRNAs) and RNA binding proteins (RBPs) of 3 key genes (Li et al., 2014; Karagkouni et al., 2018). The Cytoscape is an open-source software platform for visualizing complex networks (Shannon et al., 2003). In this study, we constructed miRNA and RBPs networks and cytoscape’s plugin CytoHubba was used to discover key nodes in above networks (Chin et al., 2014).

### Statistical Analysis

All of our analysis were conducted using R software version 3.6.1^10^. Since neither gene expression nor macrophage abundance obeyed a normal distribution, nonparametric Mann–Whitney test was used to compare variables between groups and spearman correlation analysis was used to analyze the correlation between genes and macrophage abundance. The threshold of significant difference was set to 0.05.

## Results

### The Abundance of Tumor-Infiltrating Immune Cells (TIICs)

**FIGURE 1:**
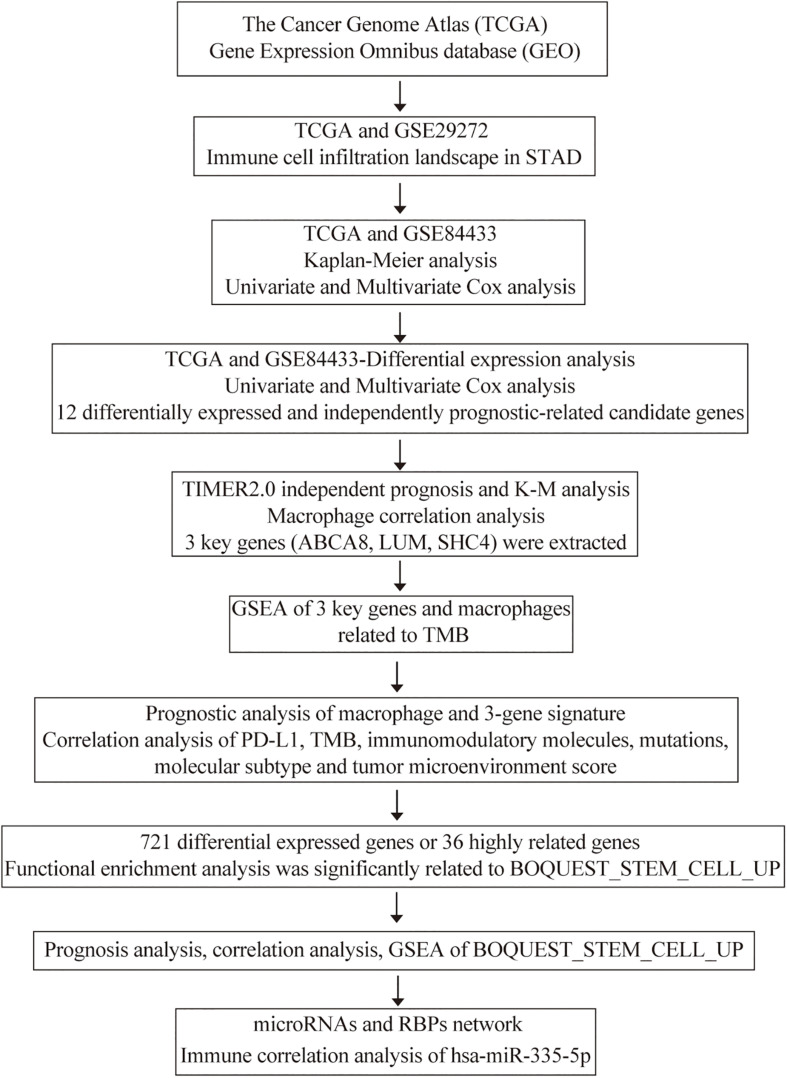
The workflow of our study.

**FIGURE 2:**
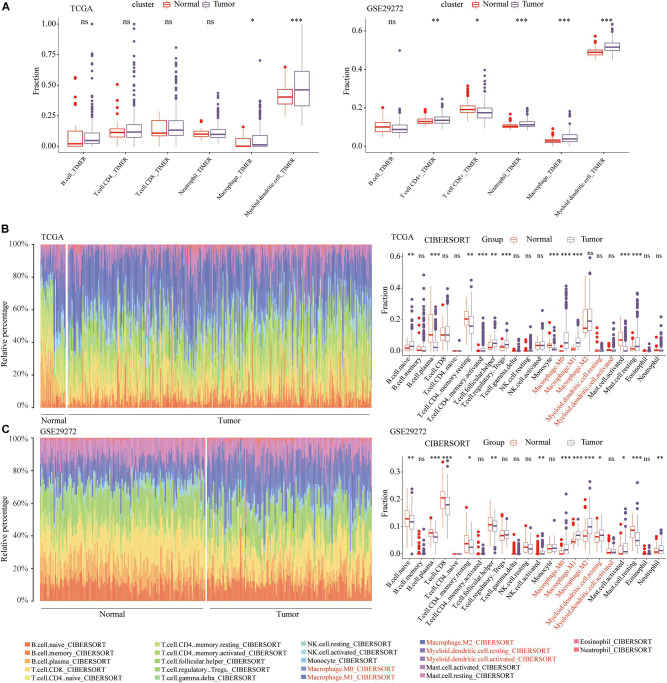
Immune cell infiltration landscape in STAD patients. **(A)** Comparison of the abundance of six types of immune cells between normal and STAD tissues. The relative proportion and comparison of 22 immune cell between normal and STAD tissues in the **(B)** TCGA and **(C)** GSE29272. *p < 0.05, **p < 0.01, ***p < 0.001.

The workflow of our study was illustrated in Figure 1. Numerous studies have shown that the abundance of tumor infiltrating immune cells are closely related to the prognosis of cancer patients and the efficacy of immunotherapy (Waniczek et al., 2017; Li et al., 2020c). The prognostic and therapeutic effects of tumor-infiltrating immune cells in stomach adenocarcinoma (STAD) have never been studied. Through the TIMER algorithm on the TIMER2.0 website, we quantitatively compared the abundance differences of 6 immune cell types between the STAD and normal samples, including CD4^+^ T cells, CD8^+^ T cells, B cells, neutrophils, macrophages, and myeloid dendritic cells. In the TCGA and GSE29272 data set, compared with normal samples, the abundance of macrophages and myeloid dendritic cells were significantly high in the STAD (*P* < 0.05) (Figure 2A). However, with regard to the infiltration of the other 4 immune cells, we did not draw a consistent conclusion.

To verify the results of the above TIMER algorithm, based on the CIBERSORT algorithm on the TIMER2.0 website, we further compared the abundance of macrophages and myeloid dendritic cells, including M0, M1, and M2 macrophages, resting and activated myeloid dendritic cells. In the TCGA (Figure 2B) and GSE29272 (Figure 2C), compared with normal samples, the proportions of M0 and M1 macrophages were significantly higher in STAD.

### The Relationships Between the Abundance of Macrophages and the Prognosis of STAD Patients

**FIGURE 3:**
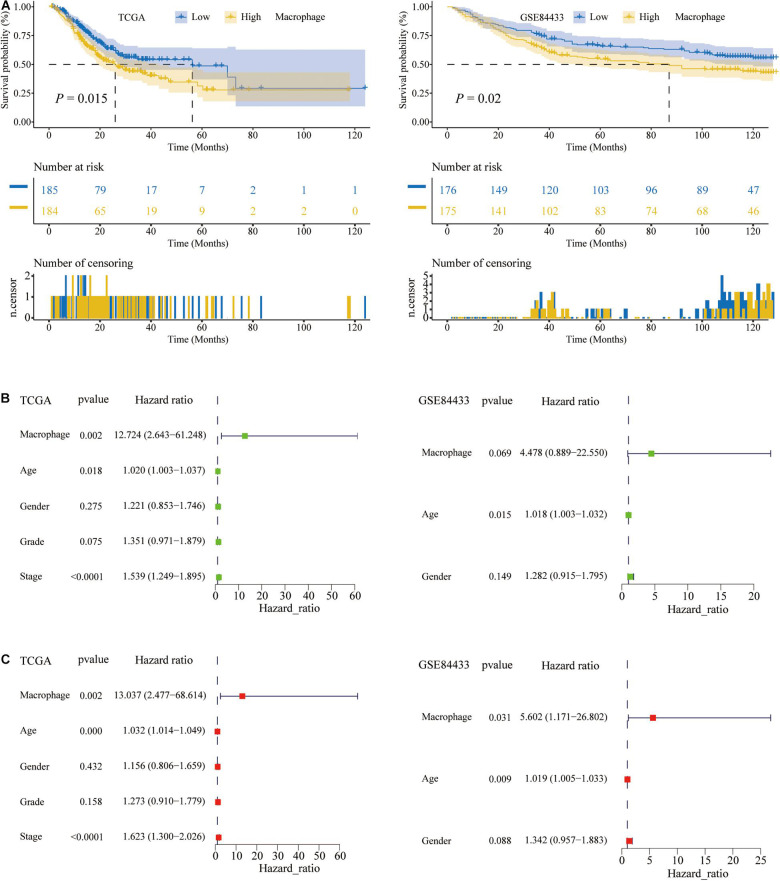
The macrophage abundance was significantly related to the prognosis of STAD patients. **(A)** The higher the abundance of macrophages, the shorter the overall survival (OS) of STAD patients. **(B)** Univariate Cox regression analysis. **(C)** Multivariate Cox regression analysis.

Previous studies had confirmed that the abundance of immune cells in the tumor microenvironment (TME) were significantly related to the prognosis of cancer patients (Pagès et al., 2009). In the TCGA and GSE84433, K–M analysis was performed to determine the association between the overall survival (OS) and the abundance of 6 immune cell types. Among 6 types of TIICs, the abundance of macrophages was significantly associated with the OS of STAD patients, and the higher the abundance of macrophages, the shorter the OS (*P* < 0.05) (Figure 3A). Besides, the abundance of the other 5 types of TIICs had no relationship with the OS of STAD patients (Supplementary Figure 1). For subpopulations of macrophages, the abundance of M0, M1, and M2 macrophage, calculated by CIBERSORT algorithm, had no relationships with the OS of STAD patients (Supplementary Figure 2). Therefore, M0, M1, and M2 macrophage could not predict the prognosis of STAD, while total macrophages were significantly related to the prognosis of STAD patients.

Given that the K–M analysis was a non-parametric test, the Cox parameter test was conducted. Also, in the above two data sets, TCGA and the GSE84433, we performed the univariate and multivariate Cox regression analysis and found that the abundance of macrophages was an independent prognostic factor for STAD patients (*P* < 0.05; Figures 3B,C). The green node represented the univariate Cox analysis, while the red represented the multivariate Cox analysis. Therefore, in STAD tissues, the abundance of macrophages was higher and related to a poor prognosis.

### Identification of Differentially Expressed Genes (DEGs) and Prognostic Related Genes Related to Macrophage Infiltrations in STAD

To evaluate the biological functions of macrophage-related genes in the occurrence and development of STAD, we systematically performed the differential expression analysis between the high and low infiltration macrophage group in the TCGA and GSE84433 data sets, respectively. 1,727 and 8,606 differentially expressed genes were extracted from the TCGA and GSE84433, respectively. The shared 1,001 differentially expressed genes in the TCGA and GSE84433 were shown in heat maps, respectively (Supplementary Figure 3A).

Based on these 1,001 genes, we performed univariate Cox analysis in TCGA and GSE84433 and obtained 214 and 237 independent prognostic-related genes, respectively. The multivariate Cox analysis was then performed on these genes, respectively, and 37 and 227 independent prognostic-related genes were extracted. Finally, we obtained 12 shared independent prognostic macrophage-related genes and displayed them in forest plots (Supplementary Figure 3B).

### The Expression and Mutation of 12 Macrophage-Related Genes

**FIGURE 4:**
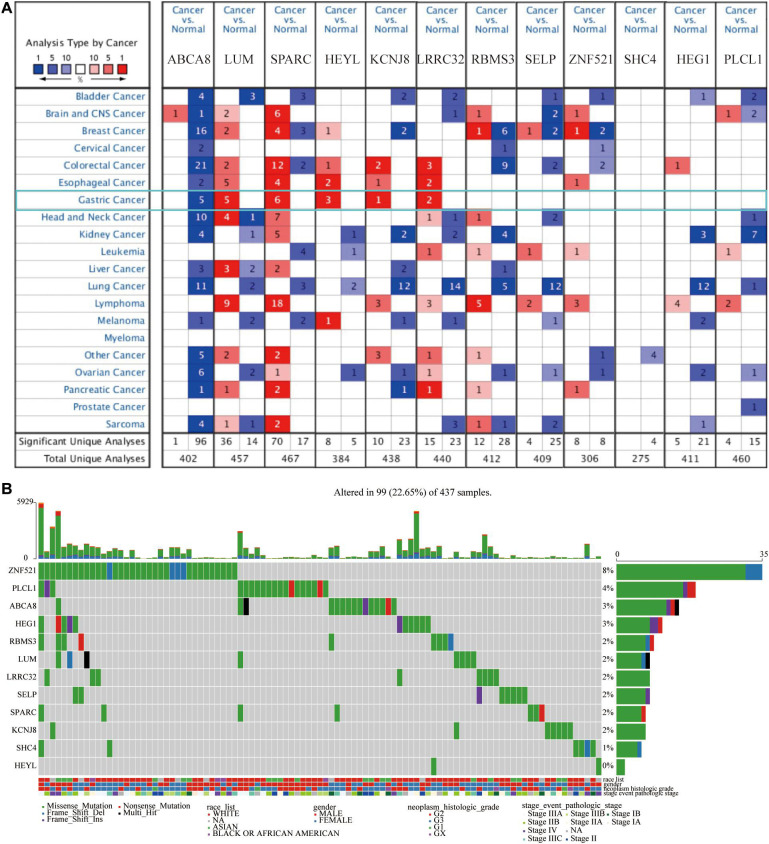
Twelve candidate genes expression and mutation analysis. **(A)** Transcriptional expression of 12 candidate genes in pan-cancer on the ONCOMINE database. **(B)** Mutation analysis of 12 candidate genes in TCGA-STAD patients.

At the RNA level, to understand the differences in the expression of 12 macrophage-related genes between pan-cancer and adjacent normal tissues, we searched the ONCOMINE database. Among these 12 genes, multiple studies had shown that the expression of ABCA8 was significantly lower, and the expression of LUM, SPARC, HEYL, KCNJ, and LRRC32 were significantly higher in STAD than normal tissues. There was no significant difference in the expression of RBMS3, SELP, ZNF521, SHC4, HEG1, and PLCL1 between STAD and adjacent tissues (Figure 4A).

Numerous studies had shown that, at the DNA level, the gradual accumulation of mutations would promote the occurrence and development of cancer (Martincorena and Campbell, 2015). Therefore, we described the somatic mutation profiles of 12 macrophage-related genes in TCGA-STAD in detail. Figure 4B showed the mutation frequency and mutation types in detail. Among above 12 genes, the mutation frequency of ZNF521 was the highest, reaching 8%, and the mutation frequency of HEYL was the lowest. Frame shift deletion was the main type of mutation.

### The Prognostic Significance of 12 Macrophage-Related Genes in STAD

**FIGURE 5:**
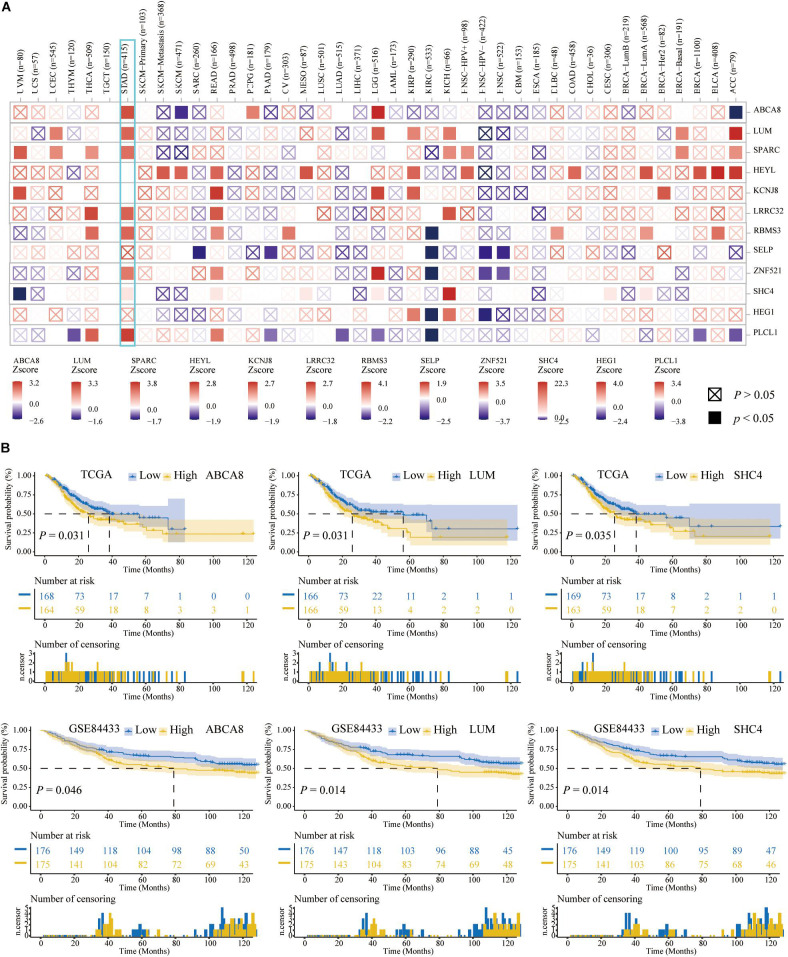
Three key genes among 12 candidate genes. **(A)** Multivariate Cox analysis adjusted the influences of age, gender, race, stages, and tumor purity on the TIMER2.0 website. **(B)** Survival curves of the 3 key genes related to the OS of STAD patients among the 12 candidate genes. The higher the expression of 3 key genes, the shorter the OS of STAD patients.

Aiming to exclude the influence of STAD patients’ age, gender, race, pathological American Joint Committee on Cancer (AJCC) stage and tumor purity on the prognosis of 12 macrophage-related genes, on the TIMER2.0 website, we performed multivariate Cox analysis. In Figure 5A, ABCA8, LUM, SPARC, LRRC32, RBMS3, ZNF521, SHC4, and PLCL1 were independent prognostic factors for STAD patients (*P* < 0.05). In addition, Figure 5A also clearly showed the prognostic performances of these 12 genes in pan-cancer.

Based on the TCGA and GSE84433, we further performed the K–M analysis and log-rank test among above 7 genes. The expression of ABCA8, LUM, and SHC4 were significantly related to the OS of STAD patients. The higher the expression, the worse the OS (Figure 5B). So far, we had captured 3 independent prognostic genes, ABCA8, LUM, and SHC4, among which there was no significant difference in the expression of SHC4 between STAD and normal tissues. For convenience, we called ABCA8, LUM, and SHC4 key genes.

### Correlation Analysis of 3 Key Genes With Macrophage Infiltrations in STAD Patients

**FIGURE 6:**
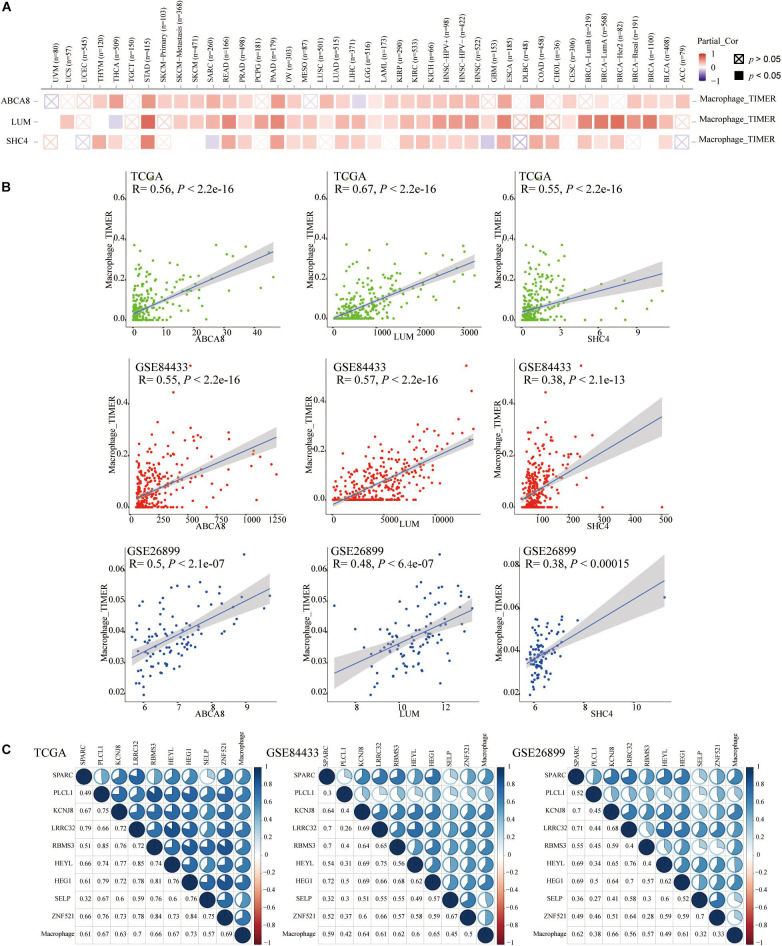
Spearman correlation analysis between 3 key genes and the macrophage abundance. **(A)** In pan-cancer on the Timer2.0, Especially in STAD, 3 key genes were significantly correlated with the abundance of macrophages. **(B)** In TCGA and GEO data sets, 3 key genes were also significantly associated with macrophage abundance. **(C)** The Spearman correlation between the other 9 genes and macrophage abundances.

On the TIMER2.0 website, we reconfirmed the correlation of 3 key genes with macrophages and found that in TCGA pan-cancer, especially in the STAD, 3 key genes were significantly associated with macrophage infiltrations (*P* < 0.05) (Figure 6A).

We once again studied the correlation between 3 key genes and macrophage infiltrations in TCGA, GSE84433 and GSE26899, in the form of a scatter plot, and got the same conclusion that 3 key genes were significantly related to the infiltrations of macrophages (*P* < 0.05) (Figure 6B). Therefore, 3 key genes were independent prognostic factors related to macrophages. In addition to the 3 key genes mentioned above, the Spearman correlation between the other 9 genes and macrophage abundances was displayed in correlograms (Figure 6C).

### Gene Set Enrichment Analysis (GSEA) of 3 Key Genes

**FIGURE 7:**
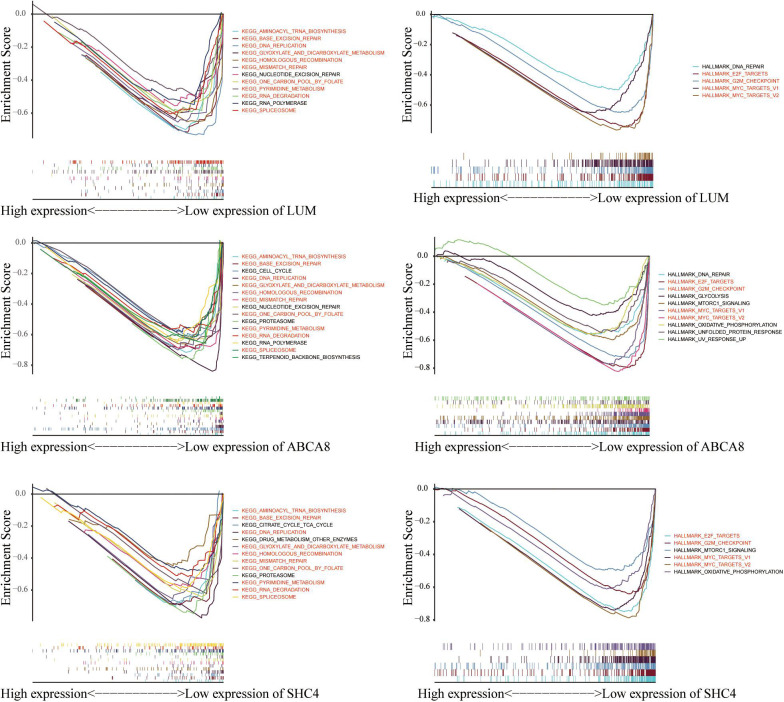
Gene Set Enrichment Analysis (GSEA) of 3 key genes. 10 KEGG pathways and 4 hallmarks were significantly enriched in the low expression of macrophage abundance and 3 key genes.

Given that the 3 key genes and macrophage abundance were independent prognostic factors for STAD, we performed GSEA to determine the shared KEGG pathways and Hallmarks between low macrophage abundance/LUM/ABCA8/SHC4 groups and high macrophage abundance/LUM/ABCA8/SHC4 groups in the TCGA-GSE84433 joint cohort. Among the results of KEGG pathways enrichment, there were 10 significantly shared KEGG pathways. Among them, “KEGG_DNA_REPLICATION,” “KEGG_BASE_EXCISION_REPAIR,” “KEGG_MISMATCH_ REPAIR,” and “KEGG_HOMOLOGOUS_RECOMBINATION” were tumor mutational burden (TMB) related pathways. For Hallmark enrichment results, there were 4 significantly shared enrichment Hallmarks. All results were shown in Figure 7, highlighted in red, as well as Supplementary Tables 2, 3. Results of GSEA suggested that 3 key genes and the abundance of macrophages might be related to the prediction of immunotherapy efficacy.

### Associations Between 3 Key Genes and Clinical Characteristics and Prognosis

**FIGURE 8:**
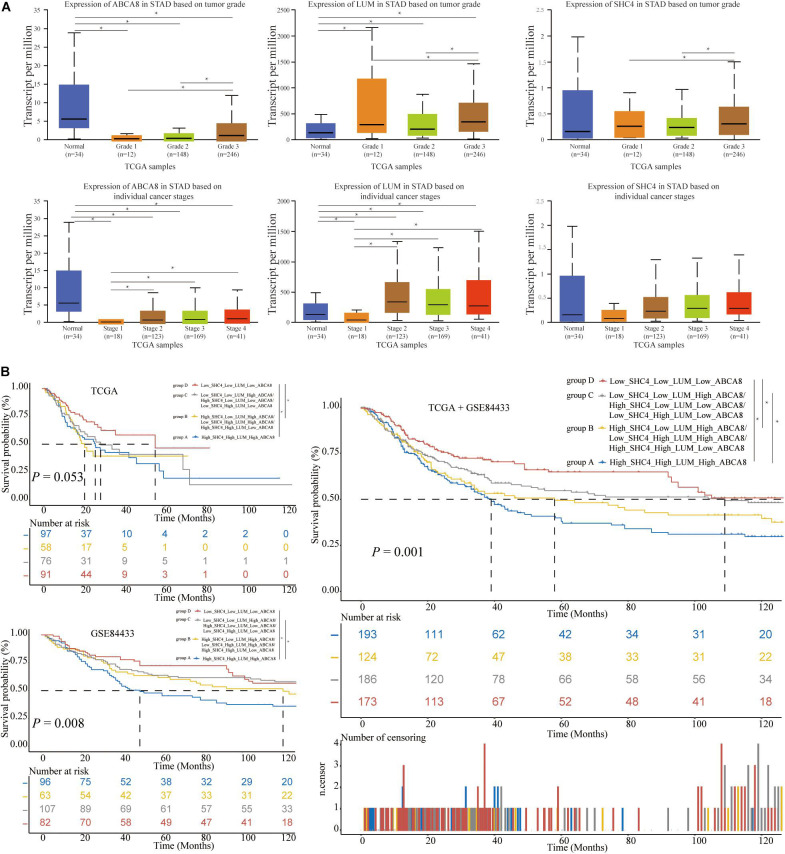
The expression of 3 key genes among different grades and stages, and the construction of 3-gene signature. **(A)** In terms of grades, the higher the STAD patient’s grade, the higher the expression of 3 key genes. In the aspect of stages, the higher the STAD patient’s stage, the higher the expression of ABCA8 and LUM. **(B)** Based on the respective medians of 3 key genes, we divided the STAD patients of TCGA and GSE84433 into 4 groups. The more up-regulated genes in 3 key genes, the shorter the OS of STAD patients. The significant difference threshold was 0.05. *p < 0.05.

For STAD grades and stages, 3 key genes were differentially expressed in different STAD grades and stages (*P* < 0.05) (Figure 8A). So far we had found that 3 key genes had similarities in prognosis, molecular pathways, regulatory networks and relevance of stages and grades. In the following research, we tried to combine 3 key genes to predict the prognosis of STAD patients and the correlation of immunotherapy.

In terms of 3 key genes jointly predicting survival, based on the respective median of 3 key genes, STAD patients were divided into 4 group, specifically, High_SHC4_High_LUM_High_ ABCA8, High_SHC4_Low_LUM_High_ABCA8/Low_SHC4_ High_LUM_High_ABCA8/High_SHC4_High_LUM_Low_ABC A8, Low_SHC4_Low_LUM_High_ABCA8/High_SHC4_Low_ LUM_Low_ABCA8/Low_SHC4_High_LUM_Low_ABCA8, and Low_SHC4_Low_LUM_Low_ABCA8. For convenience, let’s call them groups A–D. In the single TCGA data set, the OS of group D was significantly longer than that of group A and group B (*P* < 0.05). In the single GSE84433 data set, the OS of group C and group D were significantly longer than that of group A (*P* < 0.05). Combining TCGA and GSE84433, the OS of group D was significantly longer than that of groups A and B, and the OS of group C was significantly longer than that of group A (*P* < 0.05) (Figure 8B). It was not difficult to see that the more the 3 key genes were highly expressed, the worse the prognosis of STAD patients.

### The Correlation Between Macrophage Infiltrations, 3-Gene Signature, and Immunotherapy Predictors in STAD Patients

**FIGURE 9:**
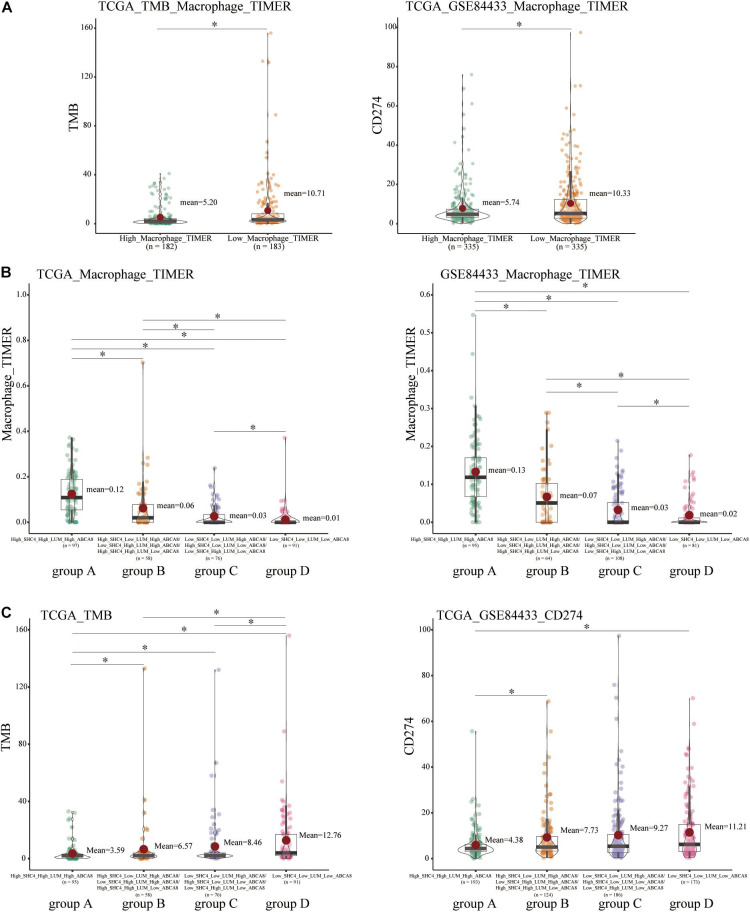
Correlation of macrophage abundance, 3-gene signature, and immunotherapy biomarkers, including tumor mutational burden (TMB) and PD-L1 (CD274). **(A)** Compared with high macrophage group, low macrophage group had higher TMB and PD-L1 expression. **(B)** There were significant differences in the abundance of macrophages in 4 groups of STAD patients divided by the 3-gene signature. **(C)** Among 4 groups of STAD patients, there were significantly differences among group A, group B, and group D on the TMB and PD-L1. *p < 0.05.

Immune checkpoint inhibitors have revolutionized cancer treatment and are approved for various cancer treatments (Duffy and Crown, 2019). Tumor mutation burden (TMB) and PD-L1 (CD274) expression are currently the main predictors of immunotherapy efficacy (Patel and Kurzrock, 2015; Chan et al., 2019). Our study revealed that the low macrophage group had higher TMB and higher PD-L1 expression (Figure 9A). We further speculated that the lower macrophage infiltrations, the better the efficacy of immunotherapy.

In terms of the combination of 3 key genes to predict the immunotherapy efficacy, since in the clinic, we could not directly score the macrophage infiltrations, and further studies show that the 3-gene signature could perfectly distinguish the macrophage abundance. For the degree of macrophage abundance, group A > group B > group C > group D (Figure 9B). The expression trends of TMB and PD-L1 in group A and D were also consistent with that of the high and low macrophage group (Figure 9C). We concluded that STAD patients in group D were most suitable for immunotherapy, while group A had the worst immunotherapy efficacy.

### The Association Between Macrophage Infiltrations, 3-Gene Signature, and Immune-Related Molecules in STAD Patients

So far we had concluded that macrophages and 3-gene signature could predict the prognosis and efficacy of immunotherapy of STAD. With the deepening of research, in addition to TMB and PD-L1 expression, more and more immunotherapy biomarkers had appeared (Danilova et al., 2019). For macrophage infiltrations and 3-gene signature, including groups A–D, there were significant differences in the expression of GZMB, EOMES, CD8A, CD4, CD276, HAVCR2, and IL-6 (*P* < 0.05) (Supplementary Figures 4A,B), which once again verified that macrophages and 3-gene signature were immunotherapy biomarkers.

### The Landscapes and Differences of Somatic Mutation of STAD

**FIGURE 10:**
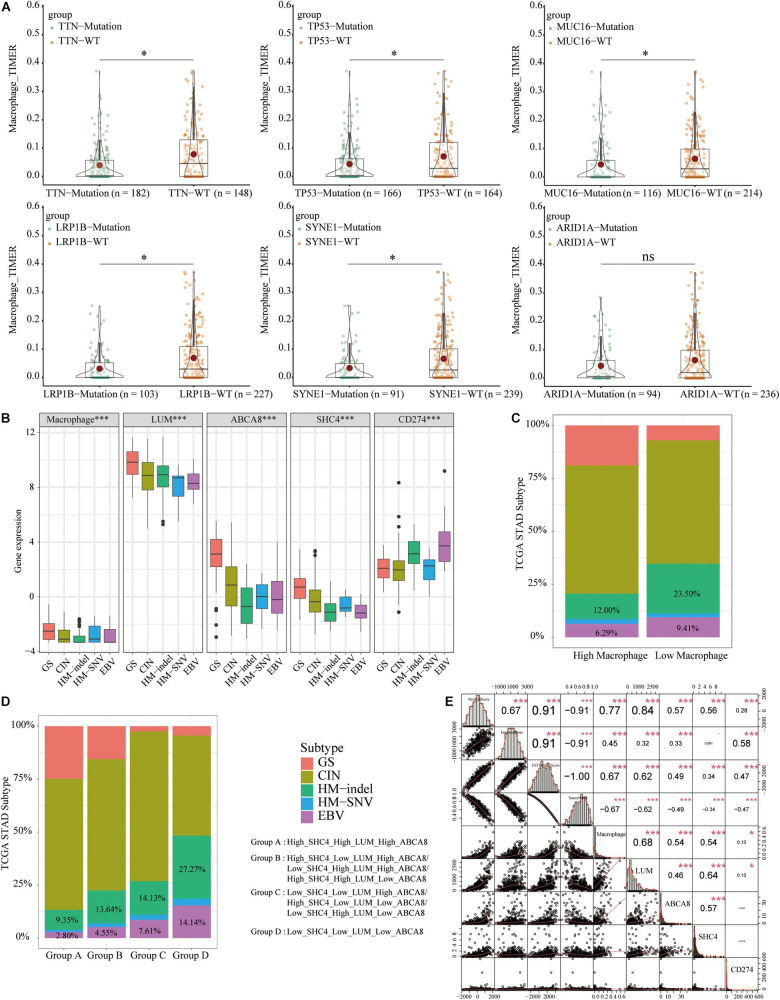
Differences in the abundance of macrophages and 3 key genes and the correlation of tumor microenvironment score. **(A)** Among the 6 most frequently mutated genes, TTN/TP53/MUC16/LRP1B/SYNE1-mutant tissues had significantly lower macrophage abundance than WT tissues. **(B)** The EBV and HM-indel subtypes had lower macrophage abundance, expression of 3 key genes and higher expression of PD-L1. **(C)** STAD tissues in the low macrophage group had a higher proportion of HM-indel and EBV. **(D)** From group A to group D, the proportion of HM-indel and EBV significantly increased. **(E)** Macrophage abundance, 3 key genes and PD-L1 were all negatively correlated with tumor purity and positively correlated with stromal scores. *p < 0.05, ***p < 0.001.

Gene mutations can cause cancer patients to be sensitive or resistant to immunotherapy, affecting the therapeutic effect (Rizvi et al., 2015). Using “maftools” package and mutation information in the cBioPortal database [Stomach Adenocarcinoma (TCGA, Firehose Legacy)], we first described the mutation of 30 genes with the highest mutation frequency of STAD (Supplementary Figure 4C). Taking the mutation frequency greater than 25% as the threshold, we extracted the top 6 genes, including TTN, TP53, MUC16, LRP1B, SYNE1, and ARID1A. Furthermore, TTN/TP53/MUC16/LRP1B/SYNE1-mutant tissues had significantly lower macrophage abundance than WT tissues (*P* < 0.05) (Figure 10A). These coincided with the impressive immunotherapy efficacy of STAD with TTN/TP53/MUC16/LRP1B/SYNE1-mutations mentioned in previous studies (Dong et al., 2017; Chen et al., 2019; Li et al., 2020a; Yang et al., 2020). It could be seen that STAD patients with low macrophage abundance could benefit more from immunotherapy.

### Molecular Subtypes and Tumor Microenvironment (TME) Score of TCGA-STAD

To develop personalized treatment for STAD patients, previous studies had divided TCGA-STAD into different molecular subtypes, including Genome stable (GS), chromosomal instability (CIN), Hypermutated- insertion and deletion (HM-indel), Hypermutated-single nucleotide variants (HM-SNV), Epstein-Barr Virus (EBV) (Liu et al., 2018). To predict the immunotherapy effects of macrophages and 3 key genes, we analyzed their expression in different subtypes and compared them with the expression of PD-L1. Among 5 molecular subtypes, GS had higher macrophage abundance and 3 key genes expression and lower expression of PD-L1. The Epstein-Barr Virus (EBV) and HM-indel had lower macrophage abundance, 3 key genes expression and higher expression of PD-L1 (Figure 10B). The EBV and HM-indel subtypes could benefit more from immunotherapy. The expression trend of PD-L1 was opposite to that of macrophage abundance and 3 key genes. STAD tissues in the low macrophage group had higher proportions of HM-indel and EBV (Figure 10C). From group A to group D, the ratio of HM-indel and EBV gradually increased (Figure 10D).

The TME scores and tumor purity were also related to the efficacy of immunotherapy (Ren et al., 2020). Macrophage abundance, 3 key genes and PD-L1 were all significantly negatively correlated with tumor purity and positively correlated with stromal and ESTIMATE scores (*P* < 0.05) (Figure 10E). We concluded that macrophages and 3 key genes could predict the immunotherapy efficacy of STAD patients. STAD patients with of low macrophage group or group D were more suitable for immunotherapy.

### Subtypes Analysis of Race and *Helicobacter pylori* (Hp) Infection

In view of the differences in clinical characteristics of different races (Asian, Black or African American, White), we studied the distribution ratio of races in 2 signatures. Among 3 races, STAD patients in low macrophage group and group D had higher proportions of Asian and Black or African American (Supplementary Figure 5A), which suggested that compared with white people, Asian and Black or African American people might be more suitable for immunotherapy.

Previous studies had shown that Hp infection affected the therapeutic efficacy of gastric cancer patients (Crowe, 2019). To understand the relationship between the Hp infection and the efficacy of immunotherapy, we explored the distribution ratio of the Hp infected and uninfected patients in 2 signatures. STAD patients in low macrophage group and groups B–D had higher proportions of the Hp uninfected patients (Supplementary Figure 5B), which indicated that the Hp uninfected patients might be more suitable for immunotherapy.

### Enrichment Analysis of High and Low Macrophage Groups and the 3-Gene Signature Groups A and D

**FIGURE 11:**
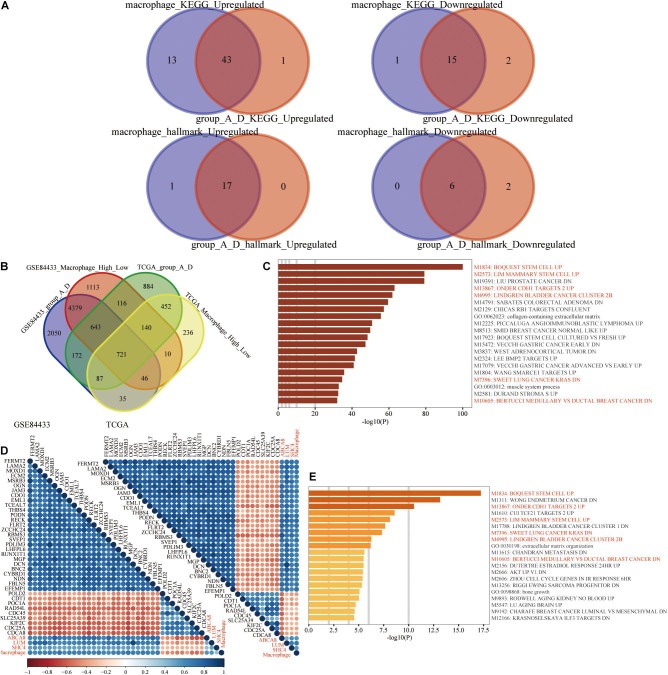
Enrichment analysis. **(A)** GSEA results of macrophage grouping and 3-gene signature grouping were mostly overlapped. **(B)** 721 differentially expressed genes (DEGs), shared by the high and low macrophage group and the 3-gene signature group A and D. **(C)** Based on the Metascape website, M1834: BOQUEST STEM CELL UP was the top functional enrichment result of 721 DEGs. **(D)** In the transcriptome data of the TCGA and GSE84433, 36 genes were highly correlated with 4 biomarkers (ABCA8, LUM, SHC4, and macrophage abundance). **(E)** M1834: BOQUEST STEM CELL UP was still the top functional enrichment result of 36 genes. Among the Top 20 enrichment results, 721 DEGs and 36 highly correlated genes had 6 identical enrichment results, which were highlighted in red.

Through the above survival analysis and predictive significance of immunotherapy efficacy, we found that there were many similarities between macrophage abundance and the 3-gene signature. The GSEA was conducted between the high and low macrophage group and the 3-gene signature group A and group D. Results of hallmark and KEGG enrichment analysis were basically the same and further proved the similarities between macrophage abundance and group A and group D. All results of GSEA were shown in Venn diagrams in Figure 11A and Supplementary Tables 2, 3.

Functional enrichment analysis was used to reveal the potential and common biological mechanisms of macrophage abundance and 3-gene signature. On the one hand, in the TCGA and GSE84433, between the high and low macrophage groups, groups A and D, differential expression analysis was performed, respectively. Based on the criteria of | log2(fold-change)| > 1 and false discovery rate < 0.05, we obtained 721 differentially expressed genes (Figure 11B). On the Metascape website, these 721 genes were mainly enriched in BOQUEST_STEM_CELL_UP (Figure 11C). Genes up-regulated in freshly isolated CD31- (stromal stem cells from adipose tissue) versus the CD31+ (non-stem) counterparts. On the other hand, we further performed Spearman correlation analysis among 19,584 protein-coding genes in the TCGA (Upper right) and 19,209 protein-coding genes in the GSE84433 (Bottom left), respectively. Based on the following thresholds, Spearman correlation coefficients with 3 key genes, and macrophage abundance >0.5 or <−0.3, we finally obtained 36 genes (Figure 11D). Similarly, these 36 genes were still mainly enriched in BOQUEST_STEM_CELL_UP (Figure 11E).

Comparing the functional enrichment results of 721 shared differentially expressed genes and 36 highly correlated genes, macrophage abundance and the 3-gene signature were all enriched M1834: BOQUEST STEM CELL UP. We speculated that the molecular mechanisms by which macrophages and 3-gene signature worked were significantly related to the genes up-regulated by stromal stem cells.

### The Prognosis and Correlation Significance of BOQUEST_STEM_CELL_UP

**FIGURE 12:**
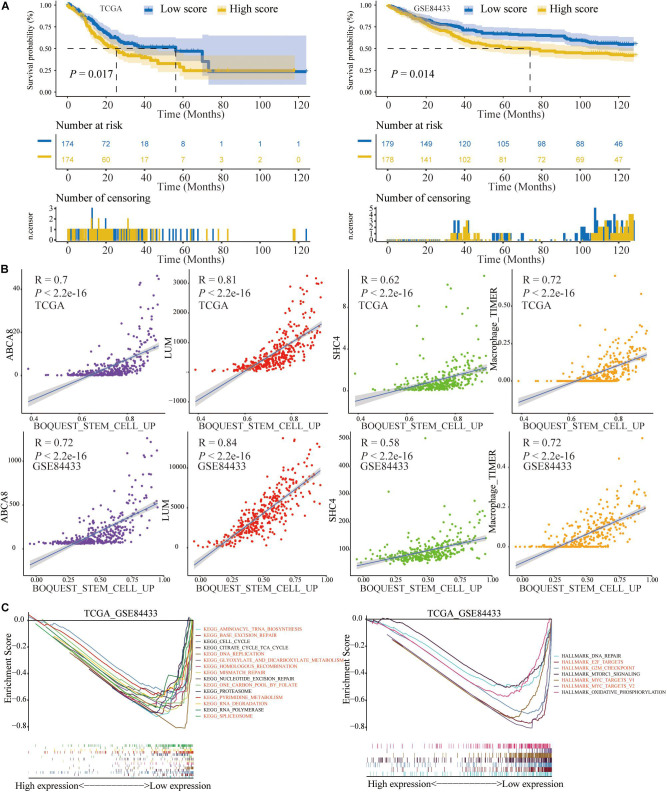
The prognosis and correlation significance of BOQUEST_STEM_CELL_UP score. **(A)** The OS of low BOQUEST_STEM_CELL_UP group was significantly longer than that of high group. **(B)** Three key genes and macrophage abundance were significantly positively related with BOQUEST_STEM_CELL_UP score. **(C)** GSEA results between high and low BOQUEST_STEM_CELL_UP group, most of which were similar with that of 3 key genes and macrophage abundance and highlighted in red.

Based on the ssGSEA method, we calculated the scores of BOQUEST_STEM_CELL_UP in single STAD sample in the TCGA and GSE84433. Like the previous 2 signatures (macrophage abundance and 3-gene signature), the OS of low score group was significantly longer than that of high score group (*P* < 0.05) (Figure 12A). After calculating the Spearman correlation, BOQUEST_STEM_CELL_UP was significantly positively correlated with macrophage abundance and 3 key genes (*P* < 0.05) (Figure 12B). Similarly, for KEGG pathways and Hallmarks, GSEA results of low score group of BOQUEST_STEM_CELL_UP were consistent with that of low expression group of macrophage abundance and 3 key genes, all of which were highlighted in red (Figure 12C). Among them, “KEGG_DNA_REPLICATION,” “KEGG_BASE_EXCISION_REPAIR,” “KEGG_MISMATCH_ REPAIR,” and “KEGG_HOMOLOGOUS_RECOMBINATION” were TMB related pathways.

### MicroRNA (miRNA) and RNA Binding Proteins (RBPs) Networks

**FIGURE 13:**
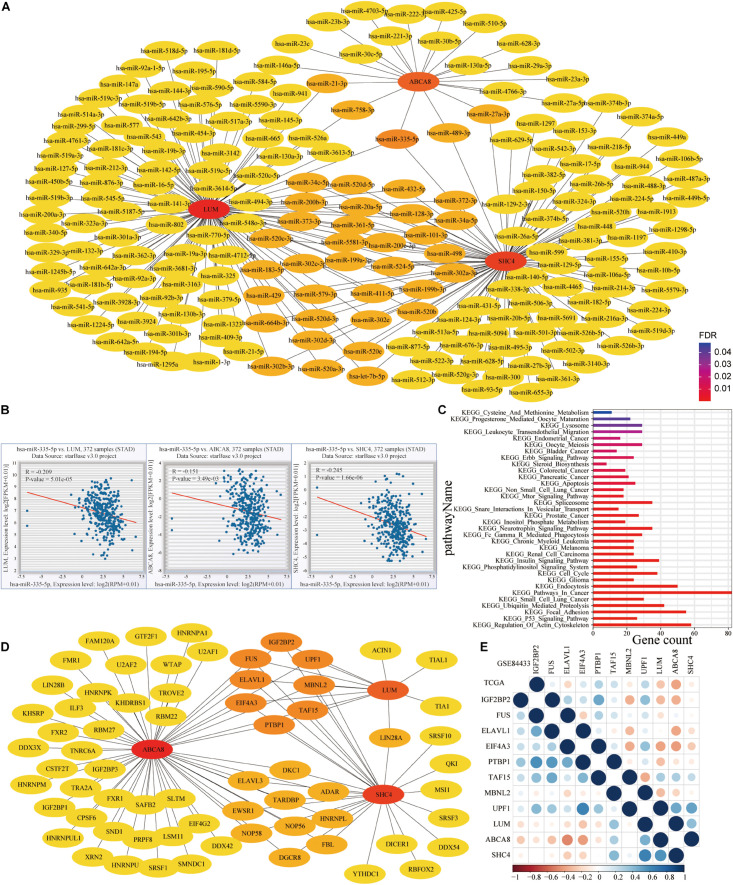
MicroRNA (miRNA) and RNA binding proteins (RBPs) networks of 3 key genes. **(A)** MiRNAs of LUM, ABCA8 and SHC4. **(B)** Hsa-miR-335-5p, shared by 3 key genes, was significantly and negatively related to them. **(C)** KEGG enrichment results for the target genes of hsa-miR-335-5p. **(D)** RBPs of 3 key genes. **(E)** Spearman correlation between 3 key genes and 8 shared RBPs in the TCGA and GSE84433.

**FIGURE 14:**
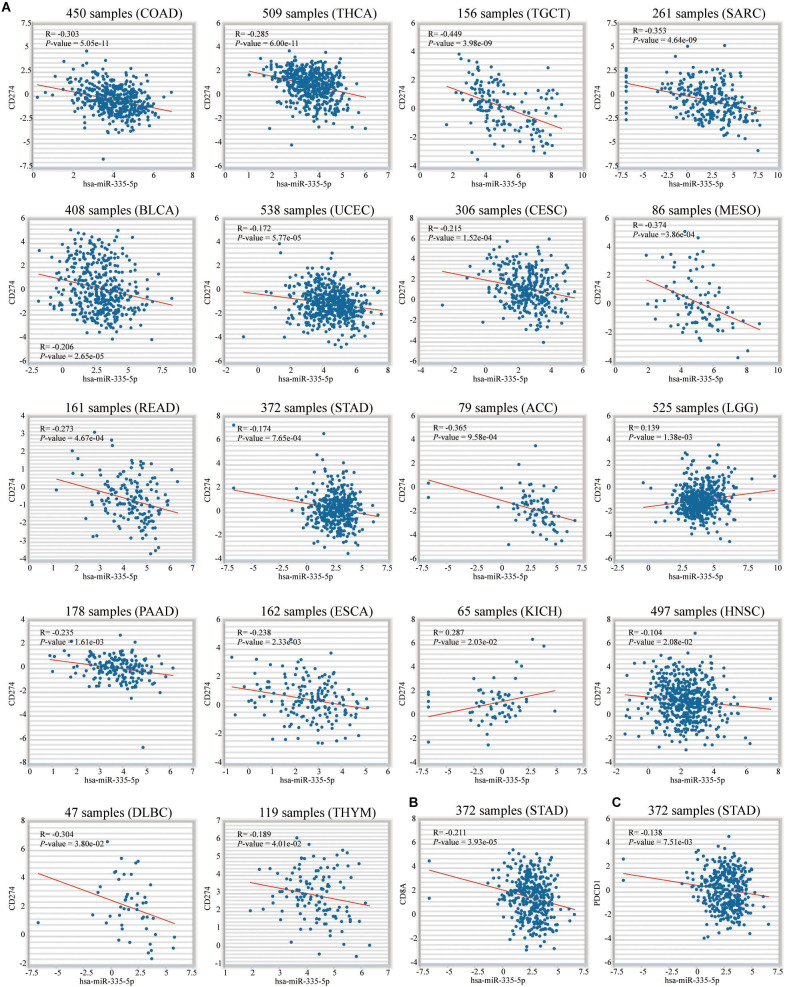
Correlation between hsa-miR-335-5p and PD-L1, CD8A and PDCD1. **(A)** Among 18 TCGA cancers including STAD, hsa-miR-335-5p was significantly associated with PD-L1. In STAD, hsa-miR-335-5p is significantly related to **(B)** CD8A and **(C)** PDCD1.

To mine the connection between 3 key genes at the level of miRNA and RNA binding protein, we conducted the following research. On the Encyclopedia of RNA Interactomes (ENCORI) website, hsa-miR-335-5p was the only miRNA shared by 3 key genes (Figure 13A). Correlation analysis also revealed that hsa-miR-335-5p was negatively related to them (*P* < 0.05) (Figure 13B). This confirmed that 3 key genes might be target genes of Hsa-miR-335-5p. Figure 13C showed results of the KEGG enrichment pathways for the target genes of hsa-miR-335-5p. We also confirmed that hsa-miR-335-5p were significantly correlated with PD-L1 in 18 types of TCGA cancer including STAD (Figure 14A). As shown in Figures 14B,C, in STAD, hsa-miR-335-5p was also significantly correlated with CD8A and PDCD1. Hsa-miR-335-5p might be a novel immunotherapy targets.

In addition, there were 8 shared RBPs, namely IGF2BP2, UPF1, MBNL2, TAF15, PTBP1, EIF4A3, ELAVL1, and FUS, respectively (Figure 13D). In the TCGA (lower left) and GSE84433 (upper right) data sets, PTBP1 was the RBPs that were significantly negatively correlated with 3 key genes, while MBNL2 was positively correlated with them (Figure 13E).

## Discussion

Stomach adenocarcinoma (STAD) is one of the most common malignancies worldwide and is also a primary cause of cancer-related mortality (Cirri and Chiarugi, 2012). Recently, numerous of novel immune checkpoint inhibitors have been proposed to improve the survival rate (Kwak et al., 2020), however, which part of STAD patients is suitable for this therapy remains to be studied.

The abundance of immune cells in the tumor microenvironment (TME) strongly influence tumor progression and the efficacy of immunotherapy. Based on the above point of view, we designed 2 similar signatures to assess the prognosis and predict the immunotherapy efficacy of STAD patients at the same time. In terms of prognostic evaluation, the higher macrophage abundance, the shorter the overall survival (OS) of STAD patients. For 3-gene signature, the higher expression of 3 key genes, the shorter the OS of STAD patients. In the aspect of predicting the immunotherapy efficacy, the lower the abundance of macrophages, the better the efficacy of immunotherapy. STAD patients of the low macrophage group and group D were more suitable for immunotherapy and had a longer overall survival (OS).

Functional enrichment analysis and the ssGSEA algorithm showed that molecular mechanisms of 2 signatures for predicting prognosis and immunotherapy efficacy were significantly related to M1834: BOQUEST_STEM_CELL_UP, which represented genes up-regulated in freshly isolated CD31- (stromal stem cells) versus the CD31+ (non-stem) counterparts (Boquest et al., 2005). The direct molecular interaction mechanisms of BOQUEST_STEM_CELL_UP predicting prognosis and immune efficacy urgently needed more wet experimental research.

MicroRNA (miRNA) is a class of non-coding single-stranded RNA with a length of approximately 22 nucleotides encoded by endogenous genes. We found that Hsa-miR-335-5p could simultaneously regulate the expression of 3 key genes, PD-L1, CD8A and PDCD1, indicating that up-regulating the expression of Hsa-miR-335-5p might be a novel biomarker. Previous research had confirmed that 2 prognostic modules of osteosarcoma were regulated by Hsa-miR-335-5p (Chen et al., 2018) and Hsa-miR-335-5p was a protective factor for rectal cancer (Slattery et al., 2015). The therapeutic and prognostic roles of Hsa-miR-335-5p in the STAD urgently needed more experimental studies.

Gene Set Enrichment Analysis (GSEA) revealed that 2 signatures were similar in molecular mechanisms. GSEA between high expression of LUM/ABCA8/SHC4/macrophage abundance/BOQUEST_STEM_CELL_UP and low of that were focused on 10 KEGG pathways and 4 hallmark gene sets. Above 14 gene sets might play a crucial role in the prognosis and immunotherapy of STAD patients.

This study constructed a 3-gene signature associated with macrophage abundance for the first time. Previous studies had proved that 3 key genes were related to the prognosis of GC patients, but the potential for predicting the efficacy immunotherapy had never been mentioned. Studies on these 3 genes were explicitly introduced as follows.

ATP Binding Cassette Subfamily A Member 8 (ABCA8) is a member of the superfamily of ATP-binding cassette (ABC) transporters. Members of the ABC1 subfamily comprise the only major ABC subfamily found exclusively in multicellular eukaryotes (Tsuruoka et al., 2002). Specifically, ABCA8 regulated lipid metabolism and participated in the formation and maintenance of myelin (Kim et al., 2013). An article on its relevance to the prognosis of GC patients had been published in 2020 (Ding et al., 2020).

Lumican (LUM) is a member of the small leucine-rich proteoglycan family, which plays the dual role of oncogene and tumor suppressor gene (Nikitovic et al., 2008). In GC, LUM plays a role as an oncogene and may be regarded as a potential prognostic indicator and treatment target for GC patients (Chen et al., 2020).

SHC Adaptor Protein 4 (SHC4) is a member of the SHC adaptor protein family. SHC family proteins are implicated in the coupling of RTK to the Ras/mitogen-activated protein kinase signaling cascade (You et al., 2010). Overexpression of SHC4 in melanoma is a prerequisite for melanoma migration and invasion. And SHC4 nuclear translocation protects melanoma cells from DNA damage caused by oxidative stress (Ahmed and Prigent, 2014). In GC, SHC4 is an independent prognostic factor (Tian et al., 2020).

To further determine which types of macrophages were related to the prognosis of STAD patients, based on the CIBERSORT algorithm, we mined the correlation between M0, M1, and M2 macrophages and the prognosis of STAD patients and M0/M1/M2 macrophages alone could not predict the prognosis of GC patients. The role of macrophage subtypes M0/M1/M2 in prognosis and immunotherapy urgently needed more research.

In order to increase the credibility, our study included multiple data sets from different sources, including TCGA, GSE8444, GSE26899, and GSE29272. We had demonstrated the potential of macrophage abundance and 3-gene signature to predict the immunotherapy efficacy from multiple perspectives, such as Tumor Mutational Burden (TMB), PD-L1, immune-related liquid molecules, immune checkpoints, STAD molecular subtype analysis and gene mutation. However, the biggest shortcoming of this study was that no wet experiments verification was performed.

## Conclusion

The macrophage abundance and 3-gene signature could predict the prognosis and immunotherapy efficacy of STAD patients. Besides, the 3-gene signature made the macrophage abundance more feasible to be used in clinical practice. Hsa-miR-335-5p and BOQUEST_STEM_CELL_UP might be novel immunotherapy targets.

## Data Availability Statement

The datasets presented in this study can be found in online repositories. The names of the repository/repositories and accession number(s) can be found in the article/Supplementary Material.

## Author Contributions

PW, SC, and YL: idea and design. TY and SC: data analysis, processing, and mapping. TY: manuscript writing. TY, PW, SZ, XW, JZ, SG, ZH, and JL: manuscript revision. All authors approved the final manuscript.

## Conflict of Interest

The authors declare that the research was conducted in the absence of any commercial or financial relationships that could be construed as a potential conflict of interest.

## Publisher’s Note

All claims expressed in this article are solely those of the authors and do not necessarily represent those of their affiliated organizations, or those of the publisher, the editors and the reviewers. Any product that may be evaluated in this article, or claim that may be made by its manufacturer, is not guaranteed or endorsed by the publisher.

## Footnotes

1
http://timer.cistrome.org/

2
https://bioconductor.org/packages/release/bioc/html/DESeq2.html

3
http://www.bioconductor.org/packages/release/bioc/html/limma.html

4
www.oncomine.org/

5
http://ualcan.path.uab.edu

6
http://www.cbioportal.org/

7
http://metascape.org

8
http://starbase.sysu.edu.cn/

9
http://carolina.imis.athena-innovation.gr/diana_tools/web/index.php?r=tarbasev8/index

10
https://www.r-project.org/
