# Supplementary figures and images for: Two Similar Signatures for Predicting the Prognosis and Immunotherapy Efficacy of Stomach Adenocarcinoma Patients
Source: Front Cell Dev Biol. 2021 Aug 3;9:704242. doi: 10.3389/fcell.2021.704242 (PMC8369372; doi:10.3389/fcell.2021.704242)

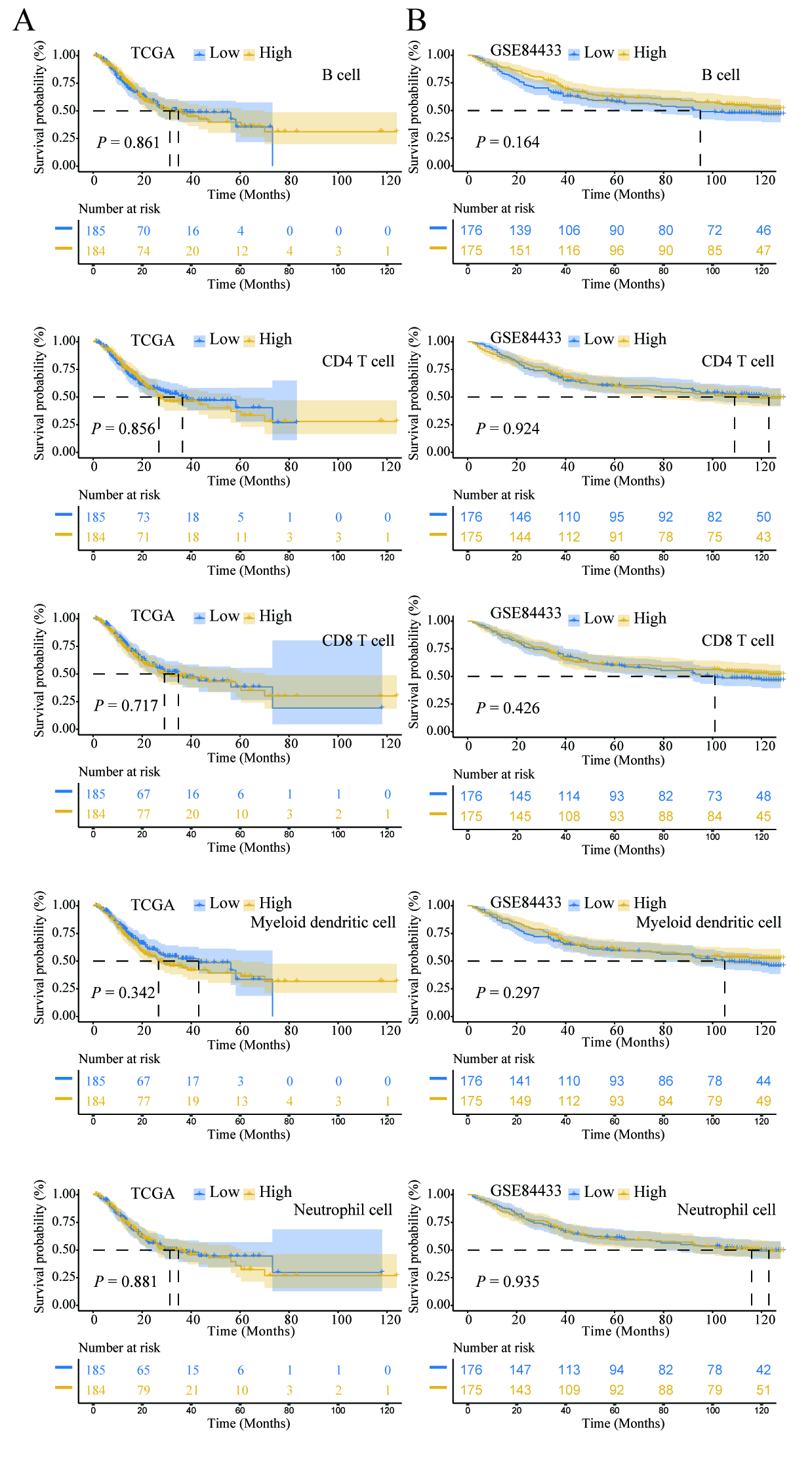

Supplement: Supplementary file 1 [file Image_1.TIF]

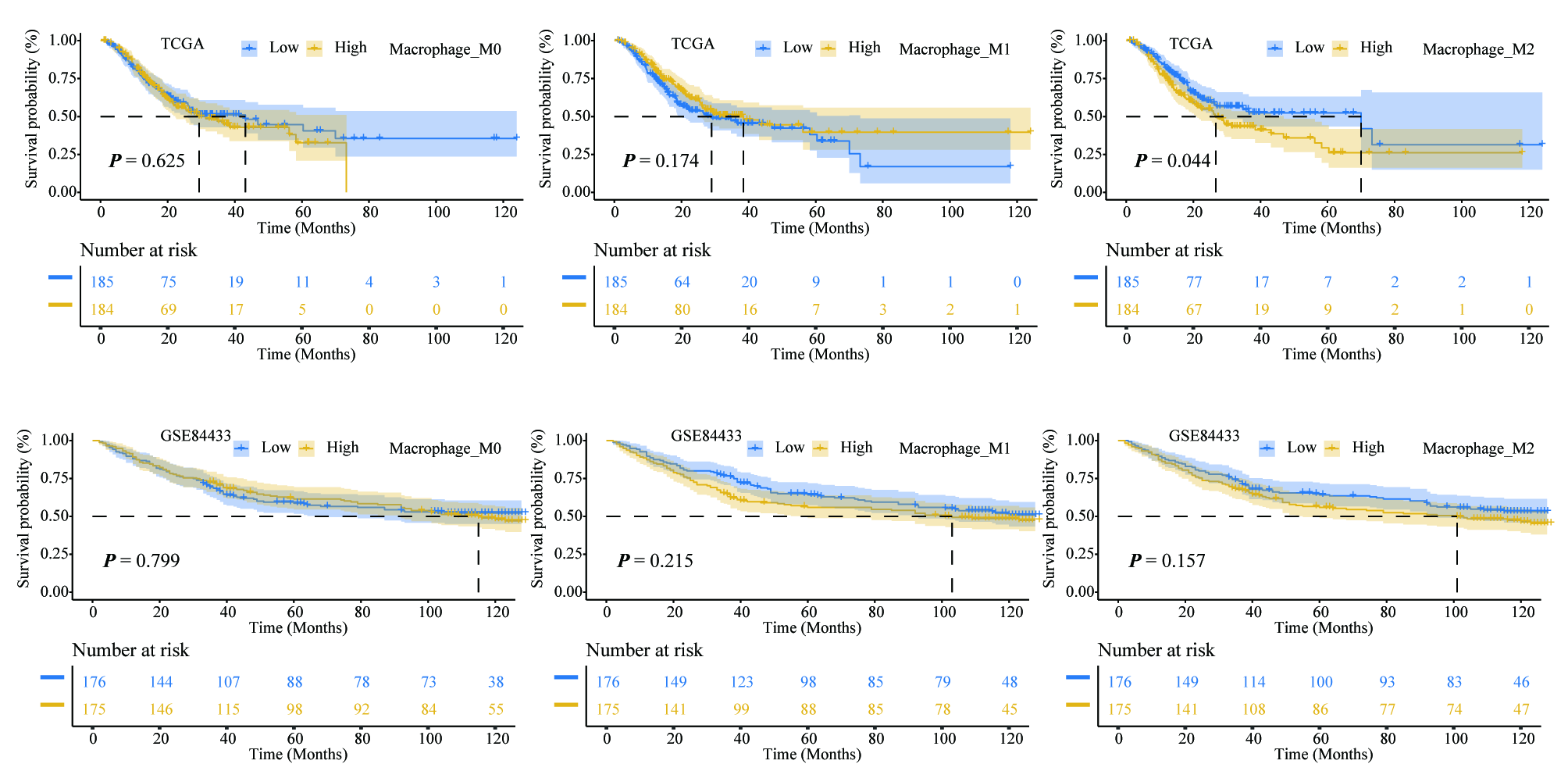

Supplement: Supplementary file 2 [file Image_2.TIF]

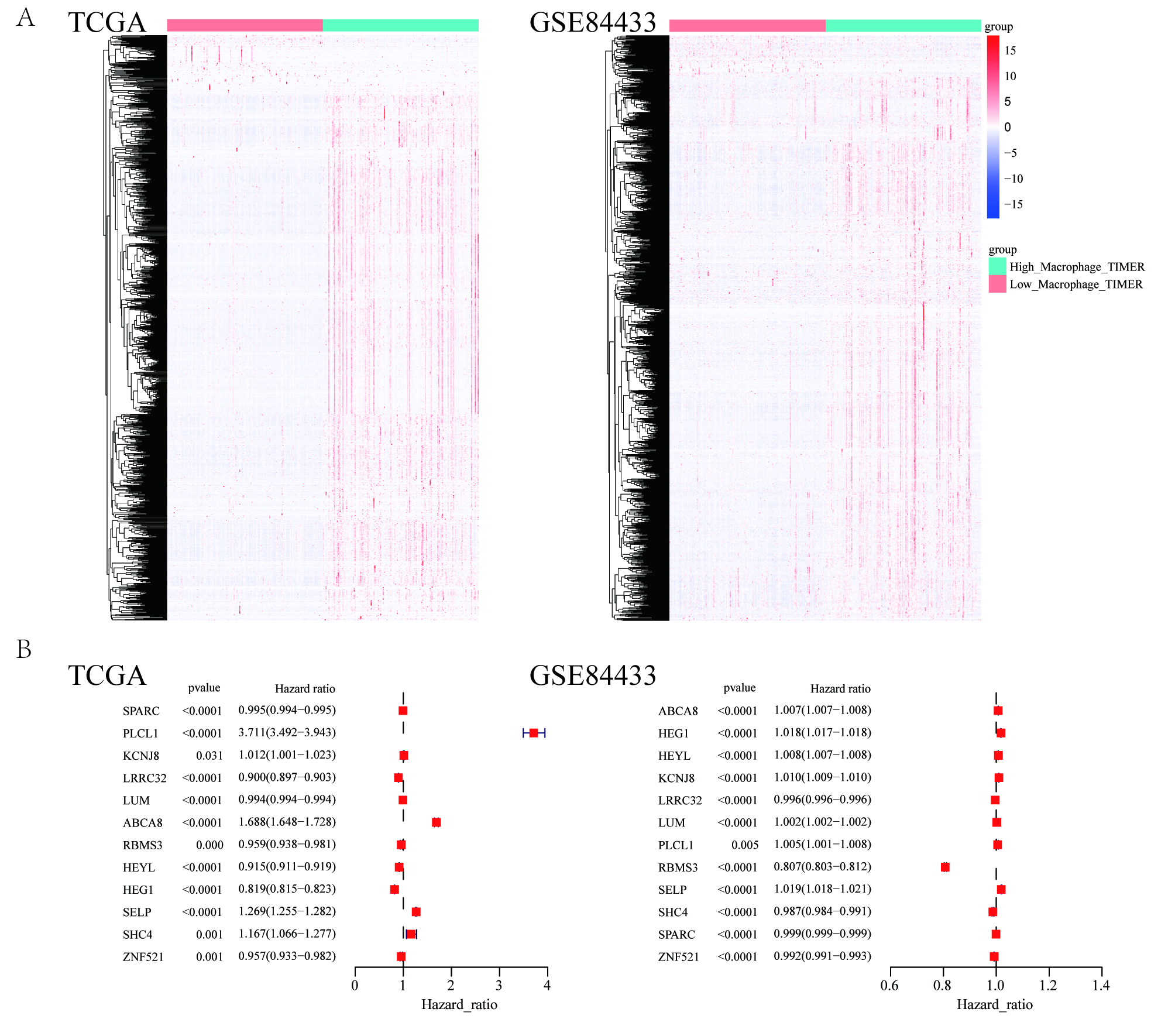

Supplement: Supplementary file 3 [file Image_3.TIF]

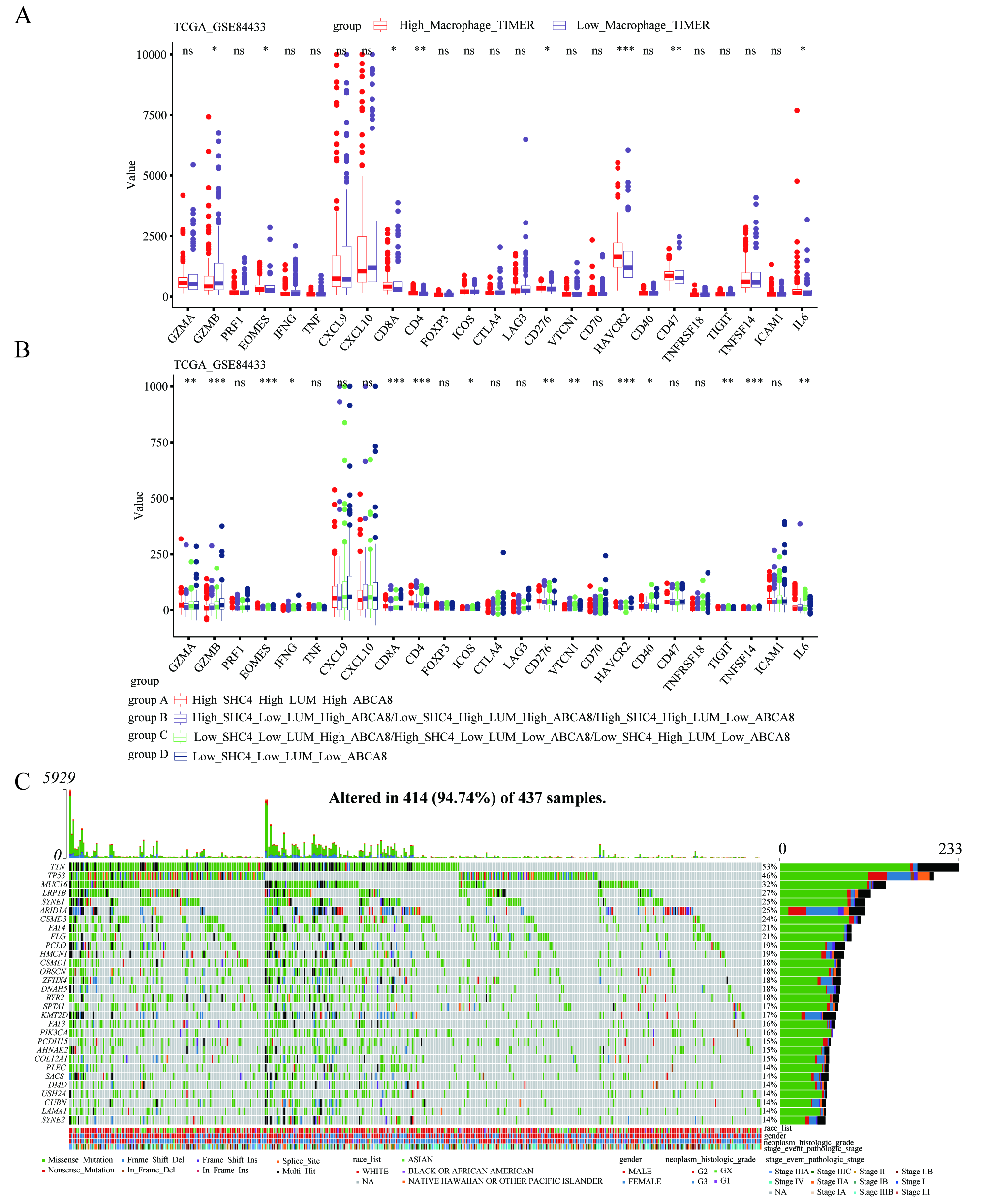

Supplement: Supplementary file 4 [file Image_4.TIF]

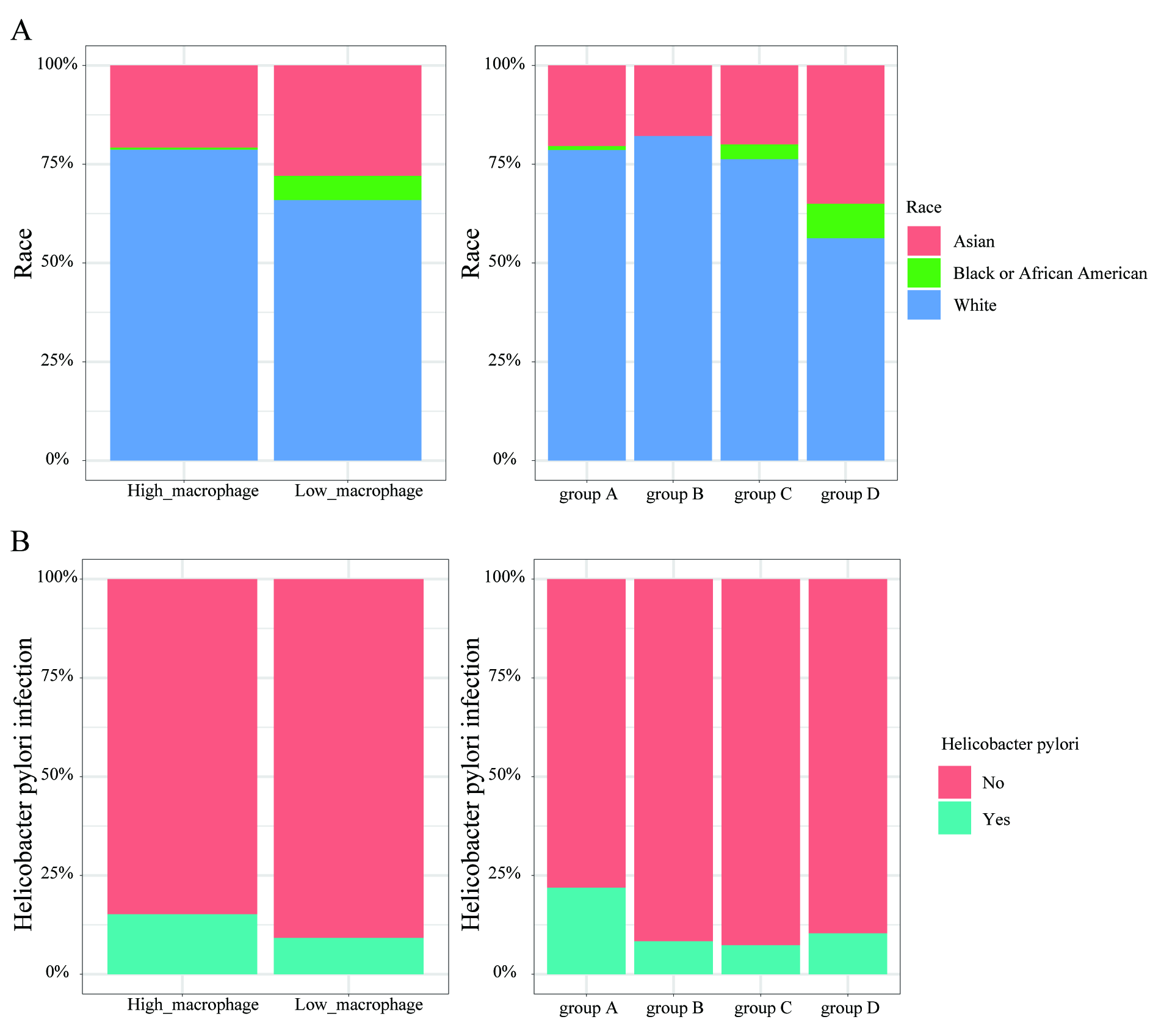

Supplement: Supplementary file 5 [file Image_5.TIF]
