# Supplementary material for: Two Similar Signatures for Predicting the Prognosis and Immunotherapy Efficacy of Stomach Adenocarcinoma Patients
Source: Front Cell Dev Biol. 2021 Aug 3;9:704242. doi: 10.3389/fcell.2021.704242 (PMC8369372; doi:10.3389/fcell.2021.704242)
Supplement: Supplementary file 6 [file Table_1.DOCX]

**Table S1. Basic information of GC patients in the TCGA and GEO database included in our study.**

| ­­­­­­ | TCGA | GSE84433 | GSE26899 | GSE29272 |
| --- | --- | --- | --- | --- |
| Normal | 32 | 0 | 12 | 134 |
| Tumor | 327 | 351 | 92 | 134 |
| Age  (median [IQR]) | 66.00  [57.50, 72.00] | 61.00  [53.00, 67.00] | 60.00  [50.00, 69.50] | NA |
| Gender =  Female / Male (%) | 118/209  (36.1/63.9) | 113/238  (32.2/67.8) | 19/73  (20.7/79.3) | NA |
| Grade (%) |  |  |  |  |
| 1 | 8 (2.4) |  |  |  |
| 2 | 110 (33.6) | NA | NA | NA |
| 3 | 209 (63.9) |  |  |  |
| Stage (%) |  |  |  |  |
| 1 | 40 (12.2) |  | 11 (12.0) |  |
| 2 | 109 (33.3) | NA | 18 (19.6) | NA |
| 3 | 146 (44.6) |  | 27 (29.3) |  |
| 4 | 32 (9.8) |  | 36 (39.1) |  |
