# Supplementary material for: Two Similar Signatures for Predicting the Prognosis and Immunotherapy Efficacy of Stomach Adenocarcinoma Patients
Source: Front Cell Dev Biol. 2021 Aug 3;9:704242. doi: 10.3389/fcell.2021.704242 (PMC8369372; doi:10.3389/fcell.2021.704242)
Supplement: Supplementary file 7 [file Table_2.DOCX]

**Table S2. KEGG GSEA results shared by macrophage abundance and 3-gene signature**

| Up-regulated KEGG terms | Macrophage_High _Low | | group_A_D | |
| --- | --- | --- | --- | --- |
|  | P-Value | FDR | P-Value | FDR |
| KEGG_CYTOKINE_CYTOKINE_RECEPTOR_INTERACTION | 0.010163 | 0.032176 | 0.003937 | 0.020481 |
| KEGG_CALCIUM_SIGNALING_PATHWAY | 0 | 0.00602 | 0 | 0.001957 |
| KEGG_LONG_TERM_DEPRESSION | 0.013972 | 0.093422 | 0.005837 | 0.065713 |
| KEGG_GLYCOSPHINGOLIPID_BIOSYNTHESIS_GANGLIO_SERIES | 0.004237 | 0.018637 | 0.00996 | 0.042856 |
| KEGG_FC_GAMMA_R_MEDIATED_PHAGOCYTOSIS | 0 | 0.03799 | 0.036822 | 0.173798 |
| KEGG_DILATED_CARDIOMYOPATHY | 0 | 0.006721 | 0 | 0.002746 |
| KEGG_PHOSPHATIDYLINOSITOL_SIGNALING_SYSTEM | 0.026263 | 0.100087 | 0.04902 | 0.177494 |
| KEGG_REGULATION_OF_ACTIN_CYTOSKELETON | 0 | 0.0158 | 0 | 0.024436 |
| KEGG_GNRH_SIGNALING_PATHWAY | 0.017857 | 0.101116 | 0.001938 | 0.064877 |
| KEGG_LEUKOCYTE_TRANSENDOTHELIAL_MIGRATION | 0 | 0.006907 | 0 | 0.013196 |
| KEGG_VASCULAR_SMOOTH_MUSCLE_CONTRACTION | 0 | 0.006914 | 0 | 0.00482 |
| KEGG_CELL_ADHESION_MOLECULES_CAMS | 0.002041 | 0.005972 | 0 | 0.009009 |
| KEGG_HYPERTROPHIC_CARDIOMYOPATHY_HCM | 0 | 0.007827 | 0 | 0.001831 |
| KEGG_ECM_RECEPTOR_INTERACTION | 0 | 0.006999 | 0 | 0.001373 |
| KEGG_MAPK_SIGNALING_PATHWAY | 0.002004 | 0.038994 | 0 | 0.059146 |
| KEGG_ADHERENS_JUNCTION | 0.022901 | 0.106135 | 0.037182 | 0.172095 |
| KEGG_GLIOMA | 0.013725 | 0.090729 | 0.028143 | 0.167946 |
| KEGG_TIGHT_JUNCTION | 0.003976 | 0.078757 | 0.007707 | 0.098149 |
| KEGG_GAP_JUNCTION | 0 | 0.026088 | 0 | 0.013693 |
| KEGG_ENDOCYTOSIS | 0.03668 | 0.121596 | 0.040541 | 0.214697 |
| KEGG_AXON_GUIDANCE | 0.003937 | 0.055695 | 0 | 0.062275 |
| KEGG_LONG_TERM_POTENTIATION | 0.00396 | 0.062067 | 0.007937 | 0.06039 |
| KEGG_COMPLEMENT_AND_COAGULATION_CASCADES | 0.001976 | 0.017721 | 0 | 0.008505 |
| KEGG_HEMATOPOIETIC_CELL_LINEAGE | 0.014374 | 0.022741 | 0.002004 | 0.014562 |
| KEGG_JAK_STAT_SIGNALING_PATHWAY | 0.004141 | 0.032896 | 0.013699 | 0.058075 |
| KEGG_ARRHYTHMOGENIC_RIGHT_VENTRICULAR_CARDIOMYOPATHY_ARVC | 0.001934 | 0.016422 | 0 | 0.004832 |
| KEGG_TGF_BETA_SIGNALING_PATHWAY | 0 | 0.030658 | 0 | 0.019136 |
| KEGG_ALDOSTERONE_REGULATED_SODIUM_REABSORPTION | 0.005917 | 0.030976 | 0.005917 | 0.042057 |
| KEGG_WNT_SIGNALING_PATHWAY | 0.009881 | 0.090464 | 0.011236 | 0.100064 |
| KEGG_CHEMOKINE_SIGNALING_PATHWAY | 0.00823 | 0.026485 | 0.007905 | 0.061132 |
| KEGG_FOCAL_ADHESION | 0 | 0.008249 | 0 | 0.003316 |
| KEGG_TASTE_TRANSDUCTION | 0.041257 | 0.081911 | 0.007737 | 0.040032 |
| KEGG_MELANOMA | 0 | 0.021738 | 0.003846 | 0.050348 |
| KEGG_MELANOGENESIS | 0 | 0.01558 | 0 | 0.007994 |
| KEGG_BASAL_CELL_CARCINOMA | 0.007937 | 0.043371 | 0.008081 | 0.042094 |
| KEGG_NEUROACTIVE_LIGAND_RECEPTOR_INTERACTION | 0 | 0.012041 | 0 | 0.003401 |
| KEGG_PATHWAYS_IN_CANCER | 0 | 0.045589 | 0 | 0.062979 |
| KEGG_NEUROTROPHIN_SIGNALING_PATHWAY | 0.006085 | 0.080439 | 0.028846 | 0.170229 |
| KEGG_VASOPRESSIN_REGULATED_WATER_REABSORPTION | 0.012448 | 0.090481 | 0.02381 | 0.109756 |
| KEGG_PROSTATE_CANCER | 0.032882 | 0.11266 | 0.024164 | 0.173324 |
| KEGG_HEDGEHOG_SIGNALING_PATHWAY | 0 | 0.017929 | 0 | 0.019896 |
| KEGG_GLYCOSAMINOGLYCAN_BIOSYNTHESIS_CHONDROITIN_SULFATE | 0 | 0.017362 | 0 | 0.019752 |
| KEGG_ARACHIDONIC_ACID_METABOLISM | 0.034623 | 0.098934 | 0.006036 | 0.041016 |

| Down-regulated KEGG terms | Macrophage_High _Low | | group_A_D | |
| --- | --- | --- | --- | --- |
|  | P-Value | FDR | P-Value | FDR |
| KEGG_RNA_POLYMERASE | 0.014228 | 0.067691 | 0.02444 | 0.087074 |
| KEGG_SPLICEOSOME | 0 | 0.049647 | 0 | 0.02224 |
| KEGG_TERPENOID_BACKBONE_BIOSYNTHESIS | 0.040169 | 0.144635 | 0.02079 | 0.085702 |
| KEGG_RNA_DEGRADATION | 0.01417 | 0.086185 | 0.002049 | 0.023972 |
| KEGG_HOMOLOGOUS_RECOMBINATION | 0.005837 | 0.048315 | 0 | 0.02091 |
| KEGG_GLYOXYLATE_AND_DICARBOXYLATE_METABOLISM | 0.040426 | 0.150615 | 0.004057 | 0.021579 |
| KEGG_MISMATCH_REPAIR | 0.006073 | 0.041596 | 0 | 0.029409 |
| KEGG_PYRIMIDINE_METABOLISM | 0.006098 | 0.040313 | 0.004032 | 0.023953 |
| KEGG_CELL_CYCLE | 0.034765 | 0.142928 | 0.004141 | 0.02662 |
| KEGG_BASE_EXCISION_REPAIR | 0 | 0.029625 | 0 | 0.023871 |
| KEGG_AMINOACYL_TRNA_BIOSYNTHESIS | 0 | 0.042135 | 0.004016 | 0.019985 |
| KEGG_ONE_CARBON_POOL_BY_FOLATE | 0 | 0.041383 | 0.007813 | 0.033411 |
| KEGG_PROTEASOME | 0.002041 | 0.037595 | 0.001988 | 0.023168 |
| KEGG_DNA_REPLICATION | 0 | 0.027245 | 0 | 0.01246 |
| KEGG_NUCLEOTIDE_EXCISION_REPAIR | 0.014286 | 0.072112 | 0 | 0.024648 |
