# Supplementary material for: Two Similar Signatures for Predicting the Prognosis and Immunotherapy Efficacy of Stomach Adenocarcinoma Patients
Source: Front Cell Dev Biol. 2021 Aug 3;9:704242. doi: 10.3389/fcell.2021.704242 (PMC8369372; doi:10.3389/fcell.2021.704242)
Supplement: Supplementary file 8 [file Table_3.DOCX]

**Table S3. Hallmarks GSEA results shared by macrophage abundance and 3-gene signature**

| Up-regulated hallmark terms | Macrophage_High_Low | | group_A_D | |
| --- | --- | --- | --- | --- |
|  | P-Value | FDR | P-Value | FDR |
| HALLMARK_HEDGEHOG_SIGNALING | 0 | 0.002414 | 0 | 0.004306 |
| HALLMARK_COAGULATION | 0 | 0.002534 | 0 | 0 |
| HALLMARK_INFLAMMATORY_RESPONSE | 0.027559 | 0.048056 | 0.026915 | 0.032208 |
| HALLMARK_KRAS_SIGNALING_DN | 0.04878 | 0.129818 | 0.021113 | 0.091639 |
| HALLMARK_COMPLEMENT | 0.008081 | 0.039831 | 0.027613 | 0.06149 |
| HALLMARK_TGF_BETA_SIGNALING | 0.039832 | 0.068074 | 0.029724 | 0.062549 |
| HALLMARK_IL2_STAT5_SIGNALING | 0.002058 | 0.012748 | 0 | 0.019749 |
| HALLMARK_MYOGENESIS | 0 | 0 | 0 | 8.78E-04 |
| HALLMARK_ANGIOGENESIS | 0.002075 | 0.002759 | 0 | 0.001533 |
| HALLMARK_HYPOXIA | 0.012422 | 0.051983 | 0.017167 | 0.066832 |
| HALLMARK_EPITHELIAL_MESENCHYMAL_TRANSITION | 0 | 6.10E-04 | 0 | 0 |
| HALLMARK_NOTCH_SIGNALING | 0.006438 | 0.043744 | 0.039419 | 0.095294 |
| HALLMARK_KRAS_SIGNALING_UP | 0.002045 | 0.002744 | 0 | 0.001652 |
| HALLMARK_APICAL_SURFACE | 0.038136 | 0.09207 | 0.018182 | 0.07159 |
| HALLMARK_APOPTOSIS | 0.006224 | 0.056275 | 0.008421 | 0.063049 |
| HALLMARK_APICAL_JUNCTION | 0 | 0 | 0 | 0 |
| HALLMARK_UV_RESPONSE_DN | 0 | 0.003168 | 0 | 0.001656 |

| Down-regulated hallmark terms | Macrophage_High_Low | | group_A_D | |
| --- | --- | --- | --- | --- |
|  | P-Value | FDR | P-Value | FDR |
| HALLMARK_MYC_TARGETS_V2 | 0 | 0.003305 | 0 | 0.010374 |
| HALLMARK_OXIDATIVE_PHOSPHORYLATION | 0.04065 | 0.043105 | 0.028986 | 0.029036 |
| HALLMARK_DNA_REPAIR | 0 | 0.011247 | 0.001938 | 0.012423 |
| HALLMARK_E2F_TARGETS | 0 | 0.002383 | 0 | 0.015207 |
| HALLMARK_G2M_CHECKPOINT | 0.006148 | 0.009181 | 0.001931 | 0.005913 |
| HALLMARK_MYC_TARGETS_V1 | 0 | 0.0024 | 0 | 0.006916 |
